# Esterification of Levulinic Acid to Ethyl Levulinate over Amberlyst-15 in Flow: Systematic Kinetic Model Discrimination and Parameter Estimation

**DOI:** 10.1021/acs.iecr.4c04540

**Published:** 2025-04-14

**Authors:** Eleni Grammenou, Maerthe Theresa Tillmann, Solomon Gajere Bawa, Arun Pankajakshan, Federico Galvanin, Asterios Gavriilidis

**Affiliations:** † Department of Chemical Engineering, University College London, Torrington Place, London WC1E 7JE, U.K.; ‡ Faculty of Mechanical Engineering, RWTH Aachen University, Elifschornsteinstraße 18, 52062 Aachen, Germany

## Abstract

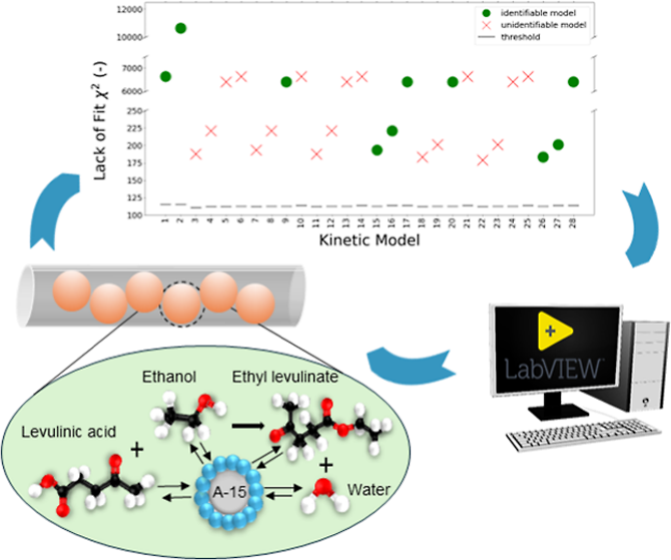

An automated reactor platform was developed using LabVIEW to conduct preplanned experiments for the identification of a kinetic model for the esterification of Levulinic acid (LA) and ethanol over heterogeneous Amberlyst-15 catalyst. A Single Pellet String Reactor of 1.25 aspect ratio was used for this kinetic study, loaded with 0.1 g of 800 μm catalyst spheres, at flow rates 20–60 μL/min, temperatures 70–100 °C, and LA feed concentrations 0.8–1.6 M. An extensive library of power law, Langmuir-Hinshelwood-Hougen-Watson and Eley–Rideal models, was screened through the application of a general procedure for model discrimination and parameter estimation. The procedure, consisting of seven steps, was applied for the investigation of different design spaces and allowed for the reformulation of models to include temperature-dependent parameters, the former leading to an increase in model identifiability and the latter resulting in enhanced model fitting. The combination of experimental data sets including the addition of the reaction product (water) in the reactor inlet stream and the incorporation of temperature dependence in the adsorption coefficients’ expression led to the identification of two suitable kinetic models out of 28 candidates (a Langmuir-Hinshelwood-Hougen-Watson and an Eley–Rideal model), both of which accounted for the adsorption of water on Amberlyst-15 and fitted the experimental data satisfactorily.

## 1 Introduction

Increasing environmental concerns drive the shift toward alternative renewable and sustainable sources as raw materials for fuel and chemical production. Lignocellulosic biomass offers sustainability and economic benefits, without disrupting the food chain, and can be converted into valuable biobased building blocks.^1^ Levulinic acid (LA) is a versatile platform chemical that can be produced through acid hydrolysis of renewable biomass, such as wheat straw^2^ and sugar cane.^3^ The possession of a ketone carbonyl group and an acidic carboxyl group imparts LA the ability to react with different functional groups to form a wide range of derivatives.^4,5^ Alkyl levulinates, produced from the esterification of LA with alcohols, have built up great momentum as fuel additives in gasoline and biodiesel^6^ and are expected to witness a rapid growth in the LA market share by 2030.^7^ Among them, ethyl levulinate (EL) exhibits good compatibility with most gasoline blends^8−10^ and has been tested in regular diesel engines, showing to improve combustion.^11^ Its sulfur-free nature and degradation via OH radicals^12^ contribute to cleaner burning,^13^ hence paving the way toward emission reductions in the transportation network.

Despite the growing interest in EL, most of the publications in its heterogeneously catalyzed synthesis deal with catalyst screening and reusability, with focus on pore geometry and acidity.^14−20^ Few kinetic studies of the esterification of LA to EL have been published in the presence of heterogeneous catalysts,^14,21−24^ with only one conducted in a continuous system,^24^ and without accounting for the interactions between the catalytic sites and the reaction mixture, described by adsorption and desorption phenomena of the participating species. It becomes, thus, clear that there is a gap in the literature regarding a consensus on the reaction mechanism of the esterification of LA to EL and the identification of reaction kinetic models that do not constitute solely empirical expressions.

Although the use of batch reactors as a tool for kinetic studies has been well established,^25^ continuous flow microreactors are an attractive alternative in synthesis and reaction optimization.^26,27^ The higher heat and mass transfer rates in microreactor systems enhance the likelihood that intrinsic reaction information is obtained that can aid process scale-up.^28^ Moreover, the ease of automation of microfluidic systems allows precise control and tuning of the reaction conditions, with minimal user intervention and maximum consumption efficiency,^29^ while their coupling with inline^30−32^ and online^33−35^ analysis tools enables the expansion of the experimental design space.

Automated flow systems for obtaining reaction kinetics have been successfully applied for homogeneous^28,36−43^ and heterogeneous (where the catalyst is solid) liquid phase,^34,44^ gas phase,^35^ and gas–liquid phase reactions.^45^ The first step to obtain a kinetic model involves identifying an appropriate kinetic model structure through the screening of a number of potential rate laws. Identification is not a trivial task in the case of heterogeneous reactions, as there are a large number of potential mechanistic models with similar structures (e.g., Langmuir-Hinshelwood-Hougen-Watson, Eley–Rideal). The identification of the final rate expression is often based on assumptions that might be difficult to verify at the macroscopic scale. This can result in similar behavior across the investigated design space, leading to poor distinguishability and necessitating the use of microscale simulation tools.^46^ In situ spectroscopic techniques, such as Raman,^47,48^ NMR,^49^ and FTIR^50−53^ can be used to probe the solid–liquid interface at the microscale; however, this can be very challenging, in the presence of bulk species and solvents with overlapping signals.^54^ The second step after deciding on the suitable model structure is to estimate with statistical satisfaction the kinetic parameters,^55^ which can often be highly correlated or practically unidentifiable in the chosen experimental design space.^56^

The aim of this work is to systematically screen an extensive library of models to obtain the kinetics of the continuous heterogeneously catalyzed esterification reaction of LA with ethanol (EtOH), starting from simple reaction rate expressions and building on mechanistically inspired models that are mathematically described by equations containing a high number of kinetic parameters. Amberlyst-15 was chosen as the solid catalyst, as it is a low-cost commercially available sulfonic ion-exchange resin^57^ with the highest activity among both zeolites and nonzeolitic materials for the esterification of LA.^14^ To ensure that reaction kinetics are not masked by external mass transfer limitations,^58^ a Single Pellet String Reactor (SPSR), where catalytic particles were packed into tubes of slightly larger diameter, was employed. SPSRs have been used for catalyst screening at industrial conditions,^59−61^ which is usually not possible in standard lab fixed-bed reactors or pilot scale units, for gas/liquid/solid systems. Considering that increasing the complexity (i.e., number of parameters) of the model structure increases the likelihood of unidentifiable parameters in the experimental design space, an automated platform with online analysis and advanced algorithms for model identification, selection, and parameter estimation was developed. Different experimental data sets (with and without product addition) were compared to investigate the impact of the different experimental design variables on kinetic model identifiability. The temperature dependence on the adsorption of different species was also incorporated into the proposed model structures.

The paper is structured as follows. In Section 2, the experimental setup is described, followed by the procedure implemented for model discrimination and parameter estimation in Section 3. The main findings are presented in Section 4, including a discussion about the effect of the design space and temperature dependence of adsorption equilibrium constants on the model adequacy and parameter precision. The shortlisted kinetic models for the synthesis of EL over Amberlyst-15 and their relevant parameters are reported, and a comparison with the existing literature is presented.

## 2 Experimental Procedure

### 2.1 SPSR

Amberlyst-15 (Sigma-Aldrich) was used as the solid catalyst for the synthesis of EL. The catalyst was dried overnight at 80 °C, sieved, and loaded inside the reactor using a vacuum pump (Edwards Vacuum). The reaction was carried out in a SPSR that consisted of a horizontally coiled poly(tetrafluoroethylene) (PTFE) tube (VWR International) with 1 mm inner diameter (ID) filled with 0.1 g of Amberlyst-15 spheres (710–850 μm sieve fraction) that comprised a reactor bed approximately 35 cm long. Inert glass beads of 425–600 μm diameter (Sigma-Aldrich) were placed upstream and downstream of the bed, extending the reactor section to 50 cm. The string of particles was held in place by a frit-in-a-ferrule filter (IDEX) at the outlet of the reactor. An important parameter in SPSRs is their low aspect ratio, defined as the ratio of the reactor diameter over the particle diameter, which is usually below 2.^62^ Due to swelling of the catalyst, the average wet diameter of the catalyst was measured by a digital microscope (VHX-600 Digital Microscope, Keyence) to be 800 μm, which resulted in a reactor aspect ratio of 1.25 (see Supporting Information, Section S1). To investigate internal mass transfer resistances, smaller Amberlyst-15 beads (500–600 μm) were loaded in a 0.75 mm ID PTFE tube (Agilent), keeping the aspect ratio similar.

### 2.2 Experimental Set-Up

**Figure 1:**
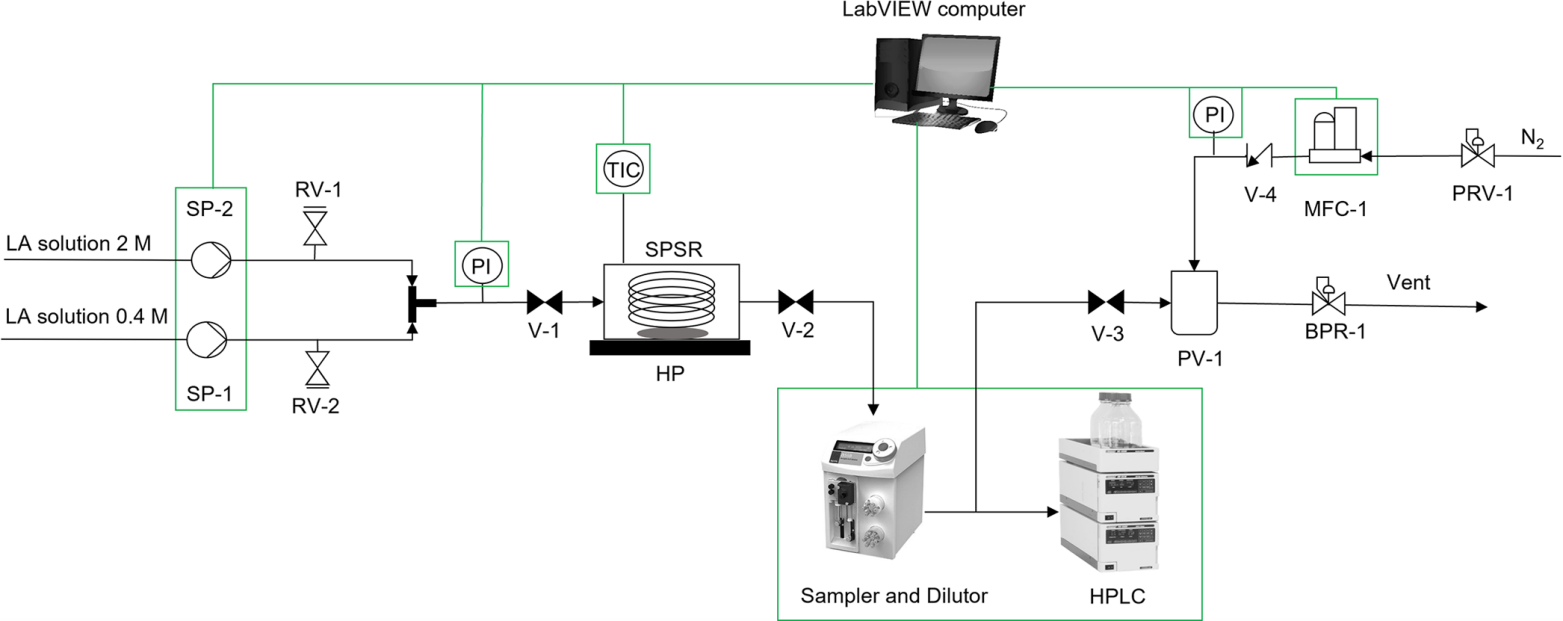
Automated platform used for the kinetic experiments of the esterification of LA to EL using Amberlyst-15 as a heterogeneous catalyst in a SPSR. Green lines indicate LabVIEW controlled or monitored equipment. SP: syringe pump, PRV: pressure reducing valve, RV: pressure relief valve, MFC: mass flow controller, PV: pressure vessel, PI: pressure indicator, BPR: back pressure regulator, V: valve, SPSR: single pellet string reactor, HP: hot plate, TIC: temperature indicator-controller, HPLC: high performance liquid chromatograph.

A schematic of the setup is shown in Figure 1. Two solutions of LA (98%, Sigma-Aldrich) in ethanol (≥99.8%, Sigma-Aldrich) with initial concentrations of 0.4 and 2 M were prepared, and each was loaded in a 10 mL stainless steel syringe (CETONI), mounted on a syringe pump (Nemesys medium-pressure module, CETONI). This enabled a range of feed concentrations by adjusting the relevant flow rates. The fluids were mixed at a T-junction of 1/16″ O.D. and 0.020 thru hole (IDEX) before entering the SPSR. The reactor was submerged into a glycerol bath, which was continuously heated and stirred by a digital hot plate (RCT 5 digital, IKA). A temperature controller, connected to the hot plate, monitored and controlled the heating fluid temperature via a partially submerged thermocouple. The reactor outlet was connected to a six-way switching valve of a sampler-dilutor (Asia Sampler and Dilutor, Syrris) equipped with a mixing chip (Micromixer Chip, Dolomite) that sent the sample into a HPLC chromatograph (LC-4000, Jasco) for the quantification of the LA and EL concentrations. Every 6 min, a sample of 10 μL of the reactor’s effluent was diluted online with the mobile phase that consisted of 60/40 acetonitrile/HPLC grade water (Sigma-Aldrich) by a factor of 100 and was injected into the HPLC column (Hypersil ODS C18, Thermo Fischer Scientific) with a flow rate of 0.8 mL/min. The column oven was maintained at 40 °C, and the detection was achieved using a UV–vis detector at 272 nm. The rest of the reaction mixture was continuously collected in a 100 mL stainless-steel pressure vessel (CRVZS-0.1, Festo). To verify that steady-state operation was reached, each experimental condition lasted for at least 3–5 residence times, with continuous HPLC sampling throughout, corresponding to a total runtime of 1 h under each set of experimental conditions. To prevent evaporation of the lower boiling components, the entire system was pressurized to 5 barg with nitrogen (BOC) using a mass flow controller (SLA mass flow controller, Brooks Instrument BV), a gas pressure sensor (40PC, 250 psig, Honeywell), and a back-pressure regulator (K series, 250 psig, Swagelok) connected to the pressure vessel. The back pressure regulator ensured that the pressure in the pressure vessel was no lower than a certain value while also working as a pressure relief valve, expelling excess gas. A liquid pressure sensor (G2500, 0–500 psi, Ashcroft) was placed upstream of the reactor entrance to measure the pressure drop in the reactor, and two pressure relief valves (U-456, 100 psi, IDEX) were installed after each syringe pump to ensure safe operation.

The experiments were fully automated using the Laboratory Virtual Instrument Engineering Workbench (LabVIEW) environment.^63^ The LabVIEW code developed in this work allowed the user to run a set of experiments by setting the temperature, feed concentration, and total flow rate at the user interface panel (see Supporting Information, Section S2). Prior to conducting the experiments, the calibration curves, based on the area of the peaks for LA and EL, were created. Since there was no driver available for the HPLC system, using its own software (ChromNAV, Jasco) and a simple Python code, the results of the analysis were sent to an Excel file, where LabVIEW was able to access and read them. In this way, the outlet concentrations of LA and EL were displayed at the end of each experimental condition. For visualization purposes, the temperature and pressure of the system were also displayed in real time in graph charts. Their maximum allowable values were set to 120 °C (maximum operating temperature of Amberlyst-15) and 6 barg, respectively. The software featured an overheating and overpressurization safety limit, which would automatically stop the experiment if these values were exceeded.

## 3 Procedure for Screening of Kinetic Models

**Figure 2:**
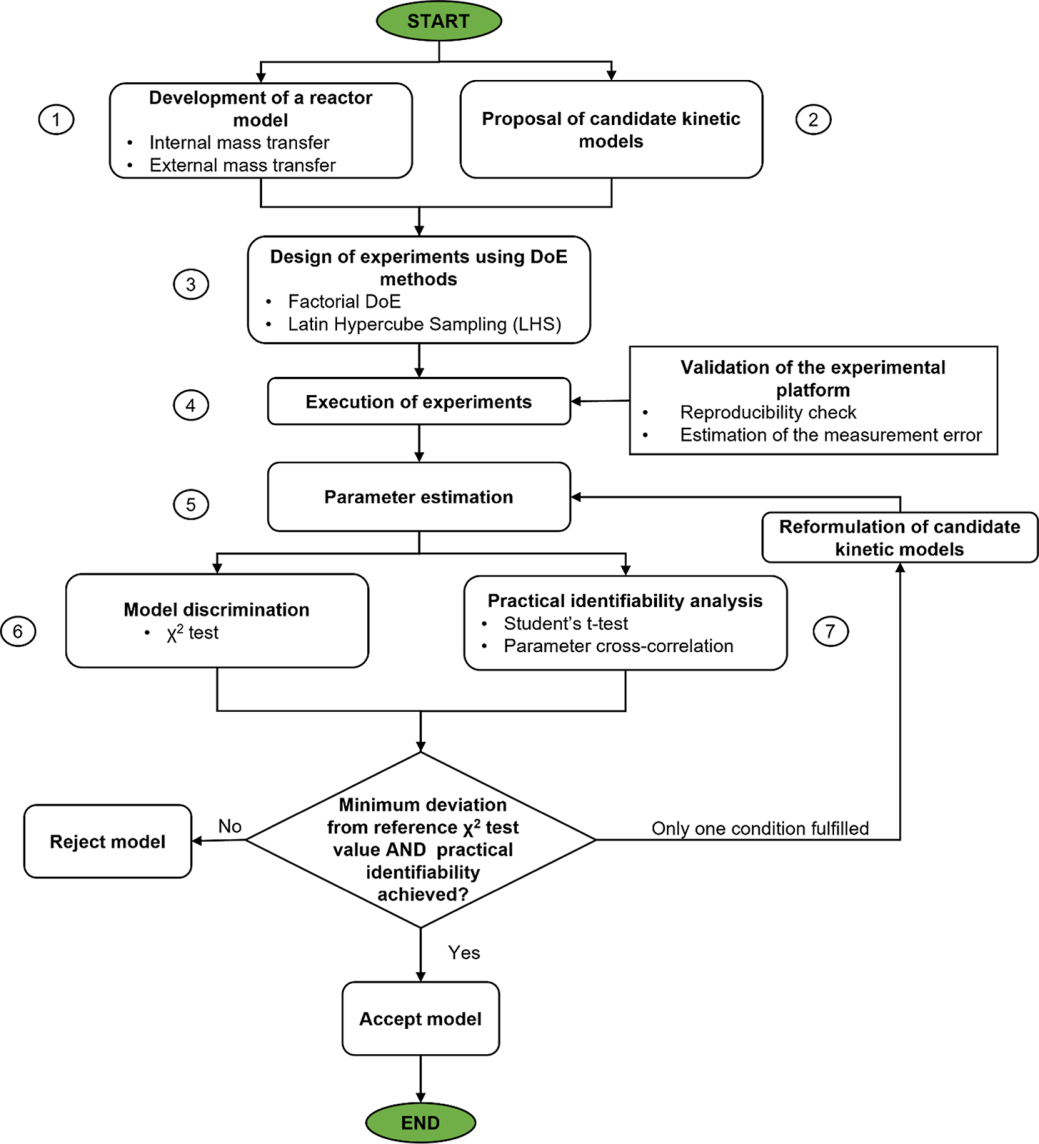
Flowchart of the general methodology proposed for kinetic model identification.

In kinetic model identification, the goal is to obtain a kinetic model that agrees with experimental data with precise estimation of the model parameters. To achieve this, the procedure followed in this work can be broken down into the steps, shown in Figure 2. A description of the steps is provided below. For further details on the applied methodology, see Supporting Information, Section S8.

1) Development of a reactor model: This step focuses on the mathematical description of the reactor used, taking into consideration external and internal mass transfer limitations.
2) Proposal of candidate kinetic models: Candidate kinetic models are proposed, which are either chosen from the literature or suggested by the user.
3) Design of experiments (DoE) using DoE methods: If there is no prior information about the reaction, a traditional DoE is performed.
4) Execution of experiments and validation of the experimental platform: The automated reaction platform is used to carry out a list of predesigned experiments provided by the user. To guarantee the platform’s robustness, a reproducibility check, as well as the estimation of the measurement error for each measurable response, is conducted.
5) Parameter estimation: Using statistical tools and the data produced from the execution of experiments, the parameters of all the proposed models are estimated.
6) Model discrimination using Lack of Fit (LoF) statistics: The fitting quality of the models is evaluated using the χ^2^ test. Any candidate model that fails the χ^2^ test is automatically rejected.
7) Practical identifiability analysis on parameter estimates: In parallel with Step 6, the models are checked for the uncertainty in the estimation of their parameters by conducting an identifiability study. If the models pass both the chi-square (χ^2^) test and all their parameters are identifiable, the procedure is complete. If the models’ parameters are unidentifiable, a reformulation of the candidate kinetic models is proposed, and the procedure returns to Step 5.

### 3.1 Development of a Reactor Model

The reactor model is a mathematical representation of the chemical reactor system. By applying principles of mass and energy conservation and incorporating reaction kinetics, it provides the concentration profiles of the various components along the reactor. Based on residence time distribution studies reported previously for a similar system,^34^ axial dispersion can be neglected. The reactor used in this study is modeled as an ideal plug flow reactor with the catalyst amount as the integration variable according to eq 1, where *m*_A15_ is Amberlyst-15 mass (g), *r*_LA_^′^ is the rate of reaction of LA per mass of catalyst (mol g^–1^ s^–1^) and *Q* is the inlet liquid flow rate (L s^–1^). Due to the size of Amberlyst-15 used, there was an indication of existence of internal mass transfer resistances.^57^ Therefore, the reaction rate in eq 1 reflects the apparent kinetics, as also verified in Section 4.1. The inlet concentration of LA in the feed (balance ethanol), temperature, and total flow rate are the control experimental variables for a constant mass of catalyst, with the concentration of LA and EL measured at the outlet of the reactor.
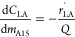
1

### 3.2 Proposal of Candidate Kinetic Models

Kinetic models express the reaction rate as a function of the concentration and temperature. For the reaction of interest, displayed in (2), candidate kinetic models are proposed (Table 1) and discriminated systematically at a later stage (Sections 3.6 & 3.7), as suggested by Steps 6 and 7 of Figure 2. These models can be derived based on the available information and assumptions made. The list of candidate kinetic models includes not only mechanistic models, which represent the potential underlying chemistry and have physical basis, but also reformulated mechanistic models (i.e., in the case of low affinity of the reaction components with the catalyst, the adsorption equilibrium constants were neglected), where reparametrization techniques have been applied^64,65^ to avoid practically unidentifiable kinetic parameters in the examined design space.
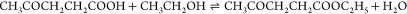
2

**Table 1:** Kinetic Models Screened for the Esterification Reaction^a^

model	rate expression/adsorption of relevant component considered				description
1	*r*_LA_^′^ = *kC*_LA_*C*_EtOH_				no adsorption on the catalytic sites^57^
2	*r*_LA_^′^ = *kC*_LA_^2^				second order with respect to levulinic acid^22^
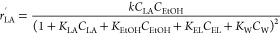					
	EtOH	LA	EL	W	
3	√	√	√	√	surface reaction between adsorbed LA and adsorbed EtOH with all species participating in the reaction rate expression^68^
4	√	√		√	weak EL adsorption (proposed)
5	√	√	√		weak H_2_O adsorption (proposed)
6	√	√			weak product desorption (proposed)
7		√	√	√	weak EtOH adsorption (proposed)
8		√		√	weak EtOH and EL adsorption^69^
9		√	√		weak EtOH and H_2_O adsorption (proposed)
10		√			strong LA adsorption (proposed)
11	√		√	√	weak LA adsorption (proposed)
12	√			√	weak LA and EL adsorption^69^
13	√		√		strong EtOH and EL adsorption (proposed)
14	√				strong EtOH adsorption (proposed)
15			√	√	strong adsorption of products (proposed)
16				√	strong H_2_O adsorption (proposed)
17			√		strong EL adsorption (proposed)
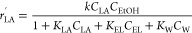					
	EtOH	LA	EL	W	
18		√	√	√	surface reaction between adsorbed LA and bulk EtOH with competitive adsorption of the products (proposed)
19		√		√	strong LA and H_2_O adsorption^70^
20		√	√		strong LA and EL adsorption (proposed)
21		√			strong LA adsorption (proposed)
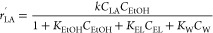					
	EtOH	LA	EL	W	
22	√		√	√	surface reaction between adsorbed EtOH and bulk LA with competitive adsorption of the products (proposed)
23	√			√	strong EtOH and H_2_O adsorption^71^
24	√		√		strong EtOH and EL adsorption^72^
25	√				strong EtOH adsorption (proposed)
26			√	√	strong product adsorption (proposed)
27				√	strong H_2_O adsorption (proposed)
28			√		strong EL adsorption (proposed)

a The tick marks indicate the corresponding species adsorption equilibrium constant being present in the mathematical formulation of the LHHW (models 3–17) or ER reaction rate expressions (models 18–28). EtOH: ethanol, LA: levulinic acid, EL: ethyl levulinate and W: water.

**Figure 3:**
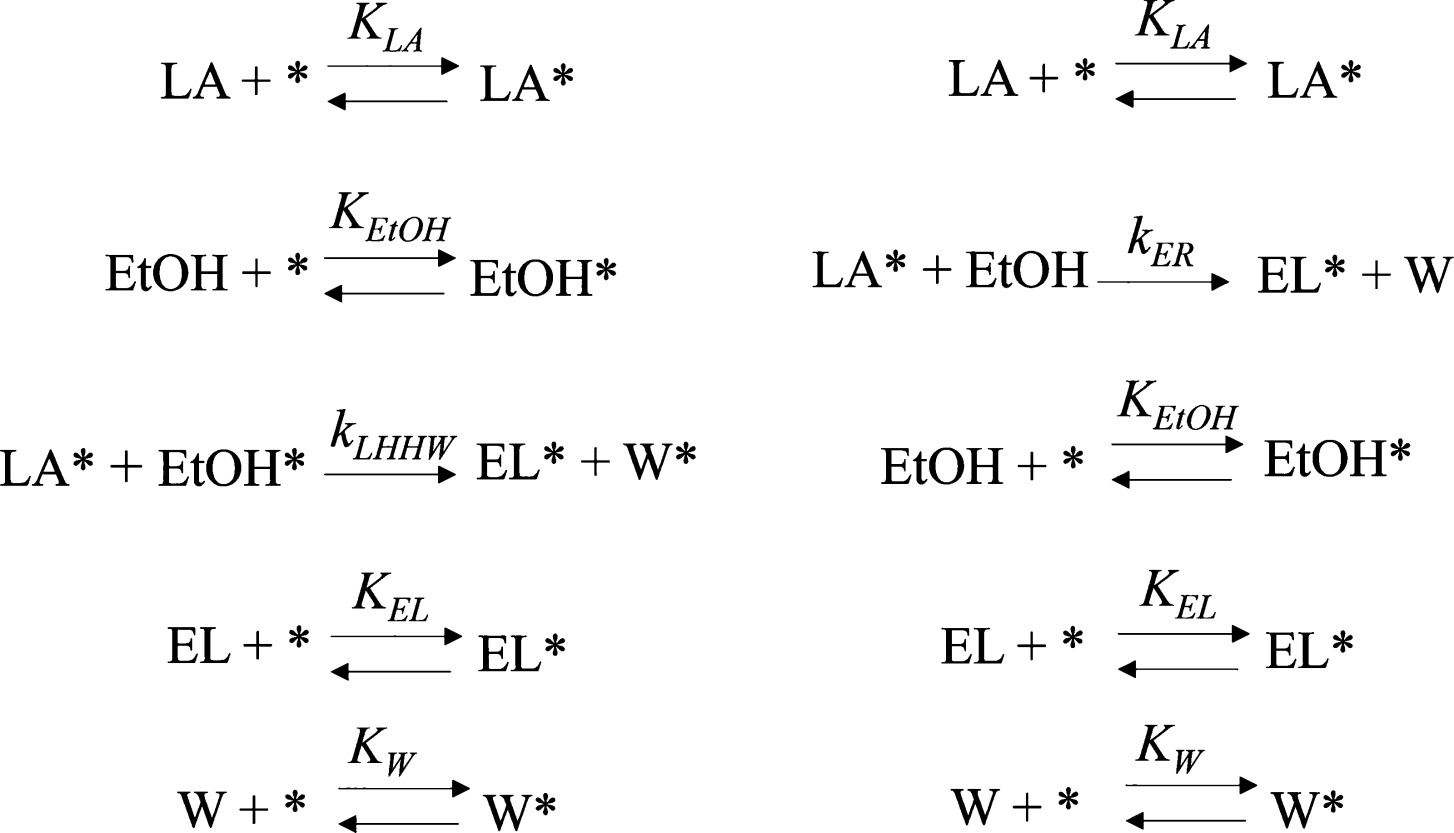
Langmuir-Hinshelwood-Hougen-Watson, LHHW (left)—and Eley–Rideal ER (right) general reaction networks as the proposed reaction mechanisms for the esterification of LA with ethanol (EtOH) forming EL and water (W) catalyzed by Amberlyst-15. (*) represents the active site.

The reaction mechanism of the esterification over ion-exchange resins revolves around the presence of sulfonic acid groups, which act as the active sites that kick-start the proton donation to the carboxylic acid.^66,67^ Unlike homogeneous catalytic systems, esterification reactions with heterogeneous solid acid catalysts, such as Amberlyst-15, involve several steps (pore diffusion, adsorption, reaction, and desorption) that happen either in parallel or sequentially, thus rendering the overall reaction mechanism (and the respective reaction rate) complex. Due to the complex interaction of the species with the catalyst active sites, esterification reactions can be described using different approaches, including pseudohomogeneous models as well as the adsorption-based Langmuir-Hinshelwood-Hougen-Watson (LHHW) and Eley–Rideal (ER) models, as shown in Figure 3. Candidate kinetic rate expressions, based on mechanistic models, were found in the literature for similar esterification systems, which, as well as other proposed formulations, were considered, as shown in Table 1. The main assumptions for all the models investigated were the following: a) The reaction was considered irreversible, due to the large excess of ethanol used (molar ratio greater than 9:1). b) The reaction on the catalytic sites was the rate-limiting step for ER and LHHW models considering adsorption, reaction, and desorption as elementary steps. c) Catalysis of the reaction from LA was not included in the kinetic expressions, as it was proven negligible for the conditions studied, as explained in Section 3.4) The reaction temperature was assumed equal to the measured temperature of the heating fluid (see Supporting Information, Section S7). e) The concentrations on the catalyst particle surface was equal to the concentrations in the bulk fluid, as there were no external mass transfer resistances (see Supporting Information, Section S6). The model was based on concentrations and not activities to avoid significant additional complexity.

For the LHHW and ER models, the kinetic rate constant, *k*, was lumped with the relevant adsorption equilibrium constants of the reactants, as this reduces correlation between the parameters.^73^ In all cases, the kinetic rate constant, *k*, was expressed in the reparametrized Arrhenius form with parameters KP_1_ and KP_2_ for the pre-exponential factor, *A*, and the activation energy, *E*_α_ (eqs 3–5).
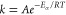
3
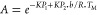
4
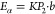
5where *T* is the reaction temperature (K), *b* is a scaling factor equal to 10,000 so that the estimated parameters are of the same order, *R* is the ideal gas constant, and *T*_M_ is the reference temperature (K) which in this work was taken as 85 °C, calculated as the average value of the upper and lower temperature limits of the experimental conditions tested.

For both LHHW and ER models, the interaction of the solid ion-exchange resin with the liquid reactants is accounted for by the adsorption constant terms *K*_LA_, *K*_EtOH_, *K*_W_, and *K*_EL_ present in the rate expressions, which are parameters to be estimated. For the esterification of LA with ethanol over Amberlyst-15, limited and often contradicting information is found regarding the adsorption strength of the investigated components on the catalyst sites,^20,57^ and no data are available regarding the range of the adsorption constants and their dependence on the reaction temperature. Adsorption experiments of nonreactive binary mixtures of components in the esterification of acetic acid with methanol revealed that component adsorption affinity onto Amberlyst-15 was augmented according to their polarity and was dependent on temperature.^74^ Therefore, water, as one of the products, was expected to adsorb the strongest on the catalyst. Similar to the mathematical formulation of the reaction rate constant, the adsorption equilibrium constant for each species, *K*_i_, can be expressed as shown in (6), where a reparametrized form of the Van’t Hoff equation, with parameters *KP*_ads1,*i*_ and *KP*_ads2,*i*_, was used to reduce the correlation between the adsorption equilibrium constant at the reference temperature, *K*_i,ref_, and the respective enthalpy of adsorption Δ*Η*_*i*_.
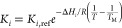
6
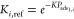
7
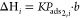
8

### 3.3 DoE

DoE methods are statistical sampling techniques that are used to achieve process knowledge, through the establishment of mathematical relationships between process inputs and outputs.^75^ Computationally cheap designs such as full or fractional Factorial Design (FD)^76^ or Latin Hypercube Sampling^77^ can be employed. Since there was limited information available about the reaction, a FD at two-levels was used, which resulted in a total of eight experimental conditions (see Supporting Information, Section S4).

Two different sets of experiments were carried out. A preliminary set of experimental conditions, denoted as Experimental Set 1, included the variation of the reaction temperature, total inlet flow rate, and inlet concentration of LA between the upper and lower limits. In all examined cases, ethanol was in excess with a molar ratio greater than 9:1. A more refined set of experiments, indicated as Experimental Set 2, was conducted with the only difference from Experimental Set 1 being the introduction of water in the feed solution at a concentration of 4 M, which guaranteed that no equilibrium was reached even at the highest temperature, based on the reaction equilibrium constant^24^ (see Supporting Information, Section S5). The reason for the introduction of water lies in the challenges associated with the distinguishability of models in the design space of Set 1, as both products are produced in equal concentrations according to the reaction stoichiometry. A detailed explanation of the influence of the design space is provided in Section 4.2.

**Figure 4:**
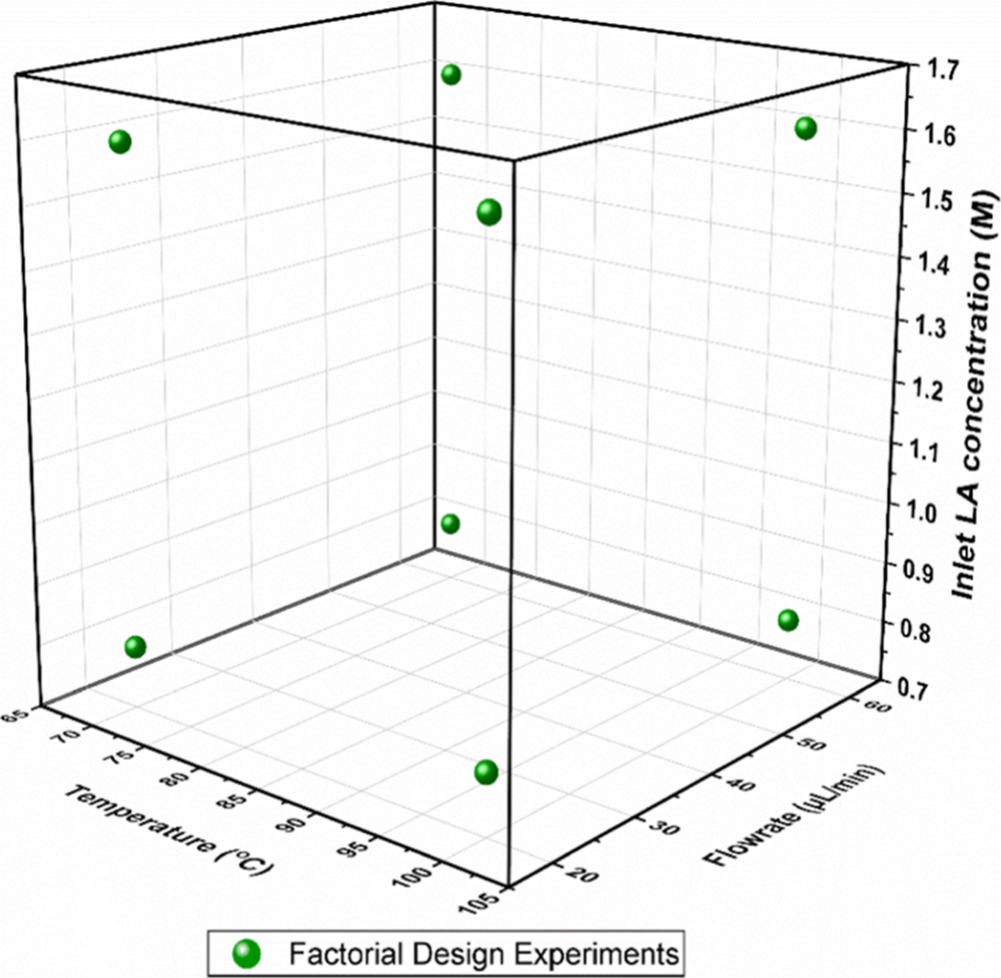
Experimental conditions of the reaction experiments designed by the factorial method.

The lower and upper limits of the input variables considered for the factorial experiments are presented in Table 2, and the design space is illustrated in Figure 4. The bounds chosen were dictated by constraints in the experimental platform. Residence times were constrained by the accuracy range of the syringe pumps, which applied pressure on 10 mL stainless steel syringes, dispensing through the system under 5 barg backpressure. The flow rates were kept in the range 20–60 μL/min to prevent the syringes emptying too quickly and to ensure that the residence time was short enough to complete an experiment within a reasonable duration. The temperature range was determined based on the heating/cooling ability of the system and the maximum operational temperature of Amberlyst-15, which was 120 °C.

**Table 2:** Experimental Control Variable Ranges Used in This Work

	experimental set 1		experimental set 2	
control variable	lower limit	upper limit	lower limit	upper limit
temperature (°C)	70	100	70	100
total flow rate (μL/min)	20	60	20	60
inlet concentration of LA (M)	0.8	1.6	0.8	1.6
inlet concentration of water (M)			4	4

### 3.4 Execution of Experiments and Validation of the Experimental Platform

For the adopted conditions, it was demonstrated by NMR spectroscopy (see Supporting Information, Section S3) that no side reactions were taking place. Moreover, given that LA can act as a catalyst for the reaction, which results in maximum 7% conversion at 90 °C after 5.5 h of batch experiments,^24^ the presence of a homogeneous reaction was investigated in the maximum temperature and lowest flow rate in an empty reactor of similar length. It was found that the amount of EL produced was negligible and could not be quantified by liquid chromatography.

The DoE campaign was conducted in three replicates. In order to establish the robustness of the system, the experimental platform was set up on two consecutive days and the reactor ran for 8 h without interference. The reproducibility of the system and the calculation of the measurements’ variance were assessed by repeating selected experiments in an identical, freshly packed reactor. The standard deviation of the measurement error for LA was calculated to be equal to 0.012 M for Set 1 (without water) and 0.021 M for Set 2 (with water), corresponding to experimental errors of 2.2% and 3.4%. Similarly, for EL, the standard deviation of the measurement was 0.015 M for Set 1 (without water), giving an error of 2.4%, while for Set 2 (with water), it was calculated equal to 0.012 M, corresponding to a 2.5% error.

### 3.5 Parameter Estimation

Parameter estimation concerns the identification of the optimal values for the unknown parameters *KP*_1_, *KP*_2_ present in the Arrhenius equation (eqs 3–5) and the parameters *KP*_ads1,*i*_ and *KP*_ads2,*i*_ of the Van’t Hoff expression (eqs 6–8) that minimize the LoF. In this work, a chi-square (χ^2^) test was used as a statistical hypothesis test to quantify and compare the LoF of candidate kinetic models. Moreover, the χ^2^ value was also used for the model discrimination described in Section 3.6.

### 3.6 Model Discrimination Using LoF Statistics

Differences in the fitting of several models were evaluated by comparing their LoF using the χ^2^ value^78,79^ (eq 9).
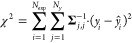
9In eq 9, *y*_*i*_ is the measured model response and  is the corresponding prediction for the *i*-th experiment for the *N*_*y*_ measurement variables, while Σ_*j,j*_ is the *jj*-th element of the variance-covariance matrix of measurement errors (see Supporting Information, Section S8.1). The LoF of each model was compared against a threshold value, also known as reference value, χ_ref_^2^, which is the χ^2^ value of the χ^2^ distribution with degrees of freedom (DoF) equal to *N*_exp_·*N*_*y*_ – *N*_θ_, where *N*_exp_ is the number of experiments, *N*_*y*_ is the number of measured variables, and *N*_θ_ is the number of unknown parameters, at a certain confidence level^80^ (here 95%). For the identification of the models reported in Table 1, the DoF ranged from 6 to 14.

In the context of this study, a less stringent criterion for the LoF was implemented, where models exhibiting a lower χ^2^ in comparison to other candidate models were still considered as potential models, even if their χ^2^ exceeded the threshold value. This relaxation of the LoF condition was motivated by the overarching objective of identifying the optimal approximation model from the pool of screened candidates.

### 3.7 Practical Identifiability Analysis on Parameter Estimates

The significance of the parameter estimates can be evaluated through the statistical Student’s *t*-test. The value of the parameter estimated by eq 10 was compared to the reference *t*-value from a Student’s *t*-distribution with *N*_exp_·*N*_*y*_ – *N*_θ_ DoF and at a significance level of 95%. If the *t*-value of a parameter is lower than the reference *t*-value, this usually indicates that the parameter is estimated poorly.^81^ The *t*-value of each parameter is defined as the ratio of the parameter estimate over the confidence interval (CI).
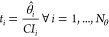
10Practical identifiability is assessed through sensitivity analysis and parameter correlation to determine whether the estimated parameters can be uniquely and accurately determined from the available data. The sensitivity analysis, calculated through the sensitivity matrix (see Supporting Information, Section S8.2), examines how variations in the parameter values influence model outputs, while the parameter correlation, quantified through the parameter correlation matrix, provides insights into parameter redundancy and uncertainty. A high correlation of parameter estimates, *C*_*i*,*j*_, and a comparable low order of magnitude of a parameter estimate are criteria for unidentifiability.^55^ In the present work, models are classified as practically identifiable if all parameter estimates and all *t*-values are higher than 0.01, for a significance level of 5%, and the parameter cross correlation, *C*_*i*,*j*_, does not exceed 0.995. If at least one of the criteria is not met, the models are considered unidentifiable.

## 4 Results and Discussion

### 4.1 Estimation of Internal Mass Transfer Resistance

Amberlyst-15 is a strongly acidic, macroreticular polymeric resin, prepared by the sulfonation of styrene copolymers cross-linked with divinylbenzene.^82^ The macroreticular pore structure of Amberlyst-15 that contains both gel and nongel porosity^83^ permits access of liquid or gaseous reactants to its active sites found throughout the bead.^84^ Due to the complex internal structure of Amberlyst-15, internal mass transfer resistances may be present when investigating reaction kinetics. Russo et al.^57^ demonstrated the presence of intraparticle diffusion limitations for the larger Amberlyst-15 particles, by examining spheres of average diameter of 600 and 300 μm, for the EL synthesis. The average particle sizes (obtained by optical microscopy) after sieving the catalyst were 755 and 550 μm, which due to swelling, resulted in diameters of 800 and 580 μm, respectively. Internal mass transfer resistances inside the 800 μm catalyst spheres were assessed using the Weisz-Prater criterion, *C*_WP_, assuming a first order reaction. For the experimental conditions studied, there was indication of internal mass transfer limitations both computationally and experimentally (*C*_WP_ > 1 at high temperatures, 100 °C, and high flow rate, 60 μL/min; see Supporting Information, Section S6).

In more complicated kinetic models, such as LHHW and ER, the calculation of the internal mass transfer resistances involves the solution of the diffusion-reaction equations.^85^ This requires that both the reaction rate constant and the adsorption equilibrium constant for all species participating in the reaction are known a priori. This is not always feasible for reactions in which there is limited information on the available kinetic models. Therefore, inclusion of internal mass transfer resistances would be overly complex. Furthermore, reduction of internal mass transfer resistances was not pursued due to constraints imposed both by the system and the catalyst. More specifically, the packing of smaller beads in a SPSR using vacuum was difficult to achieve due to pressure drop across the bed. Furthermore, bead diameters smaller than 300 μm comprised less than 1% of the commercially available catalyst.

### 4.2 Influence of Design Space on Model Identification

Models with good fit but unidentifiable parameters are flexible enough to capture the complexity of the data, but their unidentifiable parameters can take any value without significantly affecting model predictions. These unidentifiable parameters could be a result of physical phenomena that are not captured by the data set, e.g., because their effect is not measurable (such as low adsorption affinities) or due to a structural deficiency in the experimental design by overlapping and, thus, indistinguishable phenomena. The models proposed in Table 1, were therefore screened both for their adequacy to fit the experimental data as indicated by their χ^2^ value and for their parameter identifiability, as indicated by the *t*-values.

Set 1 and 2 were compared to underline the influence of different operating conditions characterizing the design space on model identification and parameter estimation. In each data set, the measurement error was separately calculated. As an initial approach, adsorption equilibrium constants for the reaction components, *K*_*i*_, were assumed temperature-independent, thus assigning a constant value, in the whole design space to decrease the complexity and computational effort required for the parameter estimation procedure. It is worth mentioning that the reaction rate constant, *k*, was always considered to be a function of temperature.

#### 4.2.1 Model Identification Using Experimental Set 1

**Figure 5:**
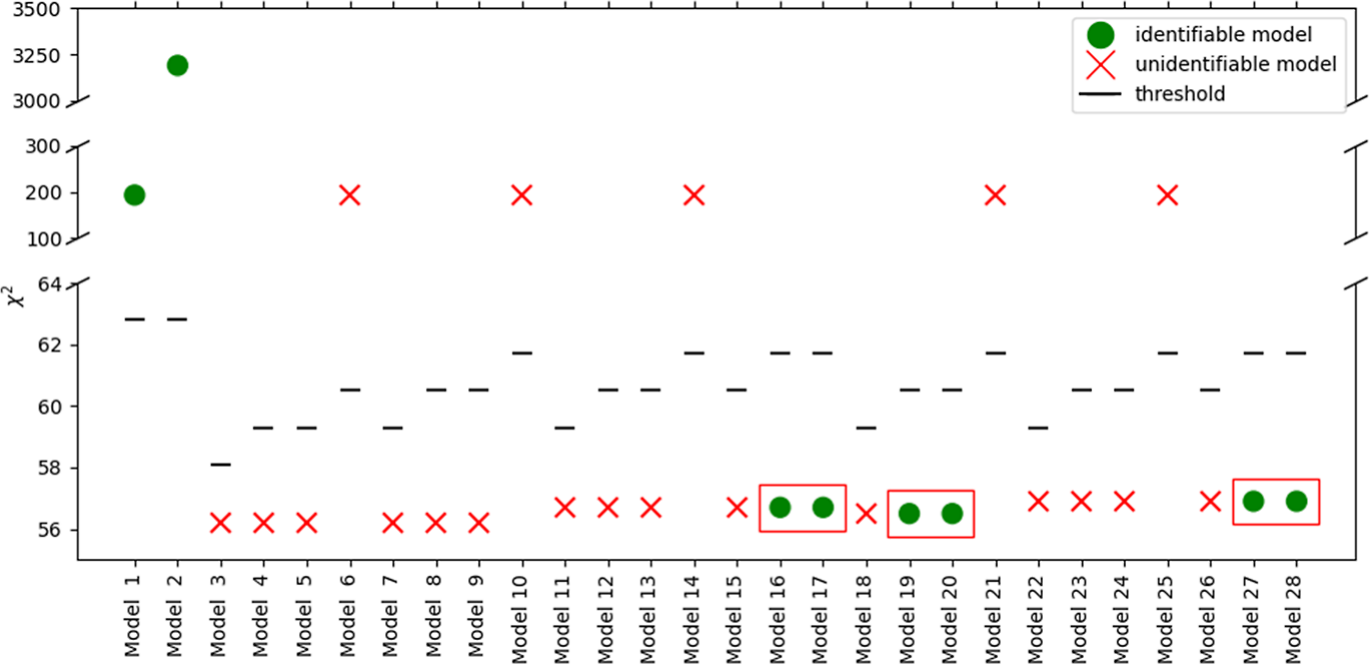
LoF metric, χ^2^, for the 28 kinetic models screened for Experimental Set 1. The red crosses indicate models with unidentifiable parameters, the green dots indicate models that are identifiable in the design space, and the red boxes indicate pairwise indistinguishability. The black dashed lines indicate the χ_ref_^2^ (threshold) value.

By applying the framework suggested in Figure 2, using the conditions of Experimental Set 1 (without water in the feed), the results of model discrimination in terms of χ^2^ statistics and practical identifiability, obtained after parameter estimation, are presented in Figure 5.

As shown in Figure 5, only 7 out of the 28 models did not pass the model identification test as their χ^2^ value was higher than the χ^2^ threshold, indicated by the dashed lines. Out of the 21 models left with χ^2^ below the threshold, 6 contained identifiable parameters, indicated by green dots. The reason behind the unidentifiability of the models that passed the χ^2^ test, when using experimental Set 1, can be attributed to the production of EL and water in identical concentrations according to the reaction stoichiometry. This leads to high cross correlation of their relevant adsorption equilibrium coefficients, *K*_EL_ and *K*_W_, and high uncertainty in their estimation. LHHW models 16 and 17 and ER models 19 and 20, as well as 27 and 28 (red boxes in Figure 5), albeit identifiable, are characterized by pairwise identical LoF values, which makes them pairwise indistinguishable. Their indistinguishability is also related to the concentration of the products, as can be seen from their denominators in the mathematical formulation in Table 1. The denominators differ only in the inclusion of either the adsorption coefficient of EL, *K*_EL_, or water, *K*_W_.

#### 4.2.2 Model Identification Using Experimental Set 2

**Figure 6:**
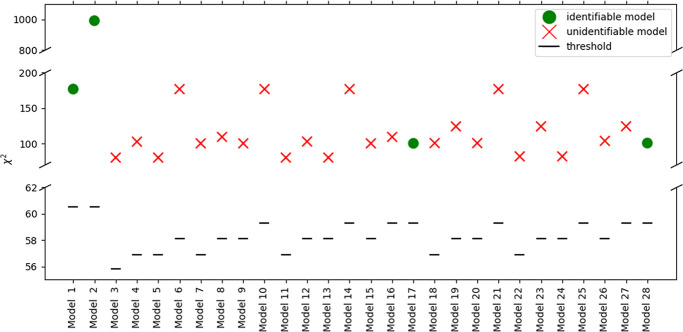
LoF metric, χ^2^, for the 28 kinetic models screened for Experimental Set 2. The red crosses indicate models with unidentifiable parameters, and the green dots indicate models that are identifiable in the design space. The black dashed lines indicate the χ_ref_^2^ (threshold) value.

The distinguishability challenges arisen from the correlation between the two reaction products when using Experimental Set 1 were overcome with the introduction of water at the inlet of the reactor to allow for differentiation of the outlet concentration for the products, introducing a new set of conditions, Experimental Set 2. The steps of the general procedure introduced in Section 3 were once again followed. In Figure 6, the results of the model adequacy and identifiability are presented for all 28 models. Removing the structural deficit associated with the reaction components’ concentrations narrowed down the identifiable models to four. However, the fitting of all of the models in the design space explored was not statistically satisfactory, as underlined by the χ^2^ test, indicating a poor fitting performance.

#### 4.2.3 Model Identification Using Both Experimental Sets

Given the results of Experimental Sets 1 and 2 separately, none of the candidate models satisfied the criteria of structural identifiability and LoF simultaneously. For this reason, the use of both data sets was attempted. It must be stated that for each response variable, the higher measurement variance from Experimental Set 1 or 2 was used as the estimated measurement error, Σ, of the blended data set.

**Figure 7:**
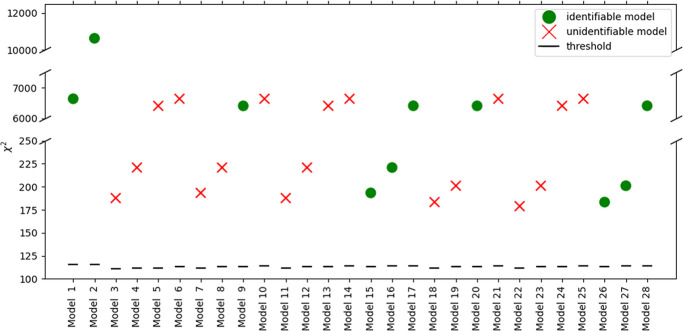
LoF metric, χ^2^, for the 28 kinetic models screened for combined Experimental Sets 1 and 2. The red crosses indicate models with unidentifiable parameters, and the green dots mark models that are identifiable in the design space. The black dashed lines indicate the χ_ref_^2^ (threshold) value.

Figure 7 presents the results of model discrimination in terms of χ^2^ statistics and practical identifiability when combining Experimental Sets 1 and 2. The combination of the two sets, with Experimental Set 2 being an expansion of the design space of Experimental Set 1, resulted in a significantly higher number of identifiable models, out of which kinetic models 15, 16, 26, and 27 showed a clearly smaller χ^2^, close to the threshold value, as compared to others. These models were the ones brought forward to the next step for improvement of model fitting by introducing a temperature-dependent formulation of adsorption equilibrium constants. The models with χ^2^ above but close to the threshold value imply adequate fitting, especially when compared to other candidate models, without fully describing the system’s complexity.

### 4.3 Model Identification Using Temperature Dependence of Equilibrium Parameters

**Figure 8:**
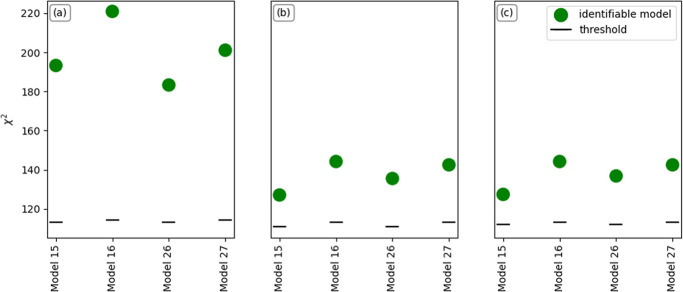
LoF metric, χ^2^, for the selected models from Section 4.2 for the combined Experimental Sets 1 and 2 (a) with constant adsorption equilibrium for water and EL, (b) including the temperature dependence of adsorption equilibrium for water and EL and (c) only including the temperature dependence of adsorption equilibrium for water but constant adsorption equilibrium for EL. The black dashed lines indicate the χ_ref_^2^ (threshold) value.

Having established the importance of combining the results of experiments conducted with (Experimental Set 2) and without the presence of water (Experimental Set 1) in the feed solution, in order to improve the fitting of the models successfully screened in Section 4.2, the incorporation of temperature in the calculation of adsorption equilibrium constants was taken into consideration, as described by eq 6. In the kinetic models 15, 16, 26, and 27, the temperature dependence of the reaction rate constant and the adsorption equilibrium constants of water and EL was accounted for, and the results of the combined data sets are shown in Figure 8. It is observed that the models’ fitting to the experimental data is improved, by comparing the χ^2^ values of Figure 8b, where the adsorption equilibrium for water and EL contains the temperature dependence, and Figure 8c, that includes the temperature dependence of adsorption equilibrium only for water, to the results of Figure 8a that were produced by assigning the adsorption equilibrium constants with a constant value, as performed in the initial approach. Furthermore, the contribution of the individual parameters on the χ^2^ is different, with *K*_W_ dependence on temperature being stronger than *K*_EL_, as seen by the negligible change in the χ^2^ value when comparing the values of Figures 8b,c.

Complementary to the evaluation of the fitting performance, the influence of the temperature on the parameter estimation was investigated. Table 3 presents the statistical precision of the parameter estimates for models 15, 16, 26, and 27, by assessing their 95% CI (see Supporting Information, Section S8) and their Student’s *t* test values (eq 10) for the two approaches followed: a) when the adsorption constants of water and EL were given a fixed value, hence the number of kinetic parameters to be estimated amounted to four, and b) when they were varied with temperature resulting in models containing six kinetic parameters. A low CI and a bigger *t*-test value than the reference *t*-value are indicative of satisfactory parameter estimation. As shown in Table 3, for the temperature-independent parameter estimates, the equilibrium adsorption constant of EL on Amberlyst-15, *K*_EL_, was not estimated with good precision for model 26, a pattern that also emerged when incorporating temperature through the Van’t Hoff equation for the same component, *KP*_ads1,EL_ and *KP*_ads2,EL_, for models 15 and 26. Therefore, based on the results of Figure 8, the expression of adsorption constants as a function of temperature improved the model adequacy, and models 16 and 27, which contained *K*_W_ as a temperature-dependent parameter, were deemed the most suitable, as they provided both the best fitting achievable (as shown in Figure 8) and reliable parameter estimates.

**Table 3:** Comparison of Parameter Estimation Results for Models 15, 16, 26, and 27 with and without Considering Temperature-dependent Adsorption Constants for Water and EL for the Combined Experimental Sets 1 and 2^a^

parameter		estimate	confidence interval [±]	*t*-value	reference *t*-value
Temperature independence					
model 15					
*KP*_1_		14.80	0.05	319.21	1.99
*KP*_2_		5.54	0.12	45.27	
*K*_W_		0.16	0.01	20.36	
*K*_EL_		0.12	0.05	2.44	
model 16					
*KP*_1_		14.90	0.02	801.69	1.99
*KP*_2_		5.34	0.09	58.16	
*K*_W_		0.15	0.01	27.58	
model 26					
*KP*_1_		14.76	0.06	262.85	1.99
*KP*_2_		5.53	0.12	45.71	
*K*_W_		0.48	0.04	12.68	
*K*_EL_		**0.25**	**0.13**	**1.87**	
model 27					
*KP*_1_		14.86	0.02	697.24	1.99
*KP*_2_		5.38	0.09	57.34	
*K*_W_		0.43	0.02	20.26	
Temperature dependence					
model 15					
*KP*_1_		14.83	0.05	314.58	1.99
*KP*_2_		5.16	0.33	15.43	
*K*_W_	*KP*_ads1,W_	1.81	0.05	36.44	
	*KP*_ads2,W_	–1.07	0.36	2.99	
*K*_EL_	*KP*_ads1,EL_	2.62	0.95	2.74	
	*KP*_ads2,EL_	**1.68**	**6.9**	**0.24**	
model 16					1.99
KP_1_		14.90	0.02	808.91	
KP_2_		4.91	0.13	37.39	
*K*_W_	*KP*_ads1,W_	1.86	0.04	49.28	
	*KP*_ads2,W_	–1.21	0.27	4.44	
model 26					
*KP*_1_		14.81	0.05	274.36	1.99
*KP*_2_		5.21	0.38	13.74	
*K*_W_	*KP*_ads1,W_	0.76	0.08	10.07	
	*KP*_ads2,W_	–1.11	0.53	2.08	
*K*_EL_	*KP*_ads1,EL_	**3.61**	**29.36**	**0.12**	
	*KP*_ads2,EL_	**13.19**	**216.91**	**0.06**	
model 27					
*KP*_1_		14.85	0.02	697.54	1.99
*KP*_2_		4.91	0.15	32.44	
*K*_W_	*KP*_ads1,W_	0.80	0.05	15.60	
	*KP*_ads2,W_	–1.42	0.37	3.83	

a The unidentifiable parameters are indicated in bold.

**Figure 9:**
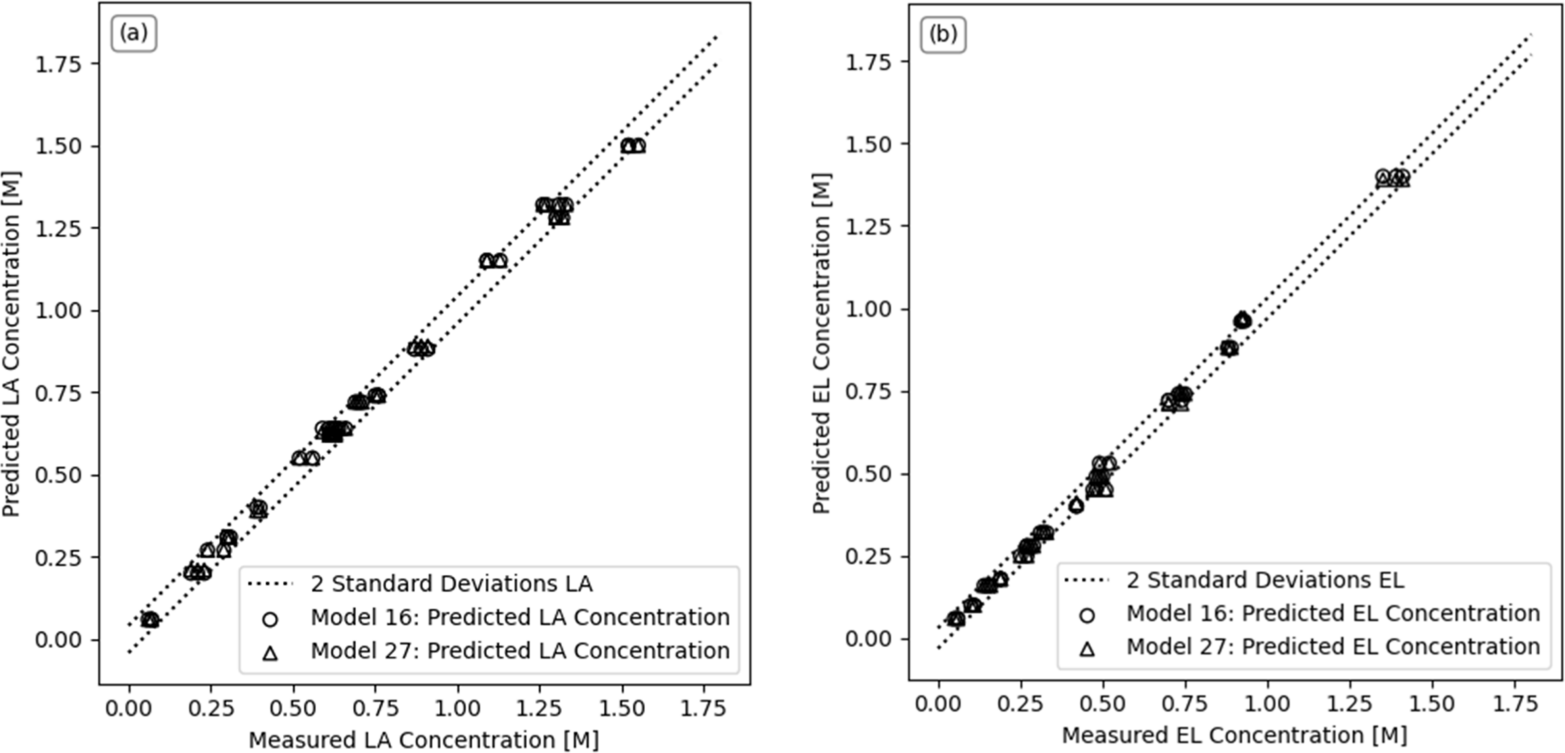
Reactor outlet concentration parity plots of the selected kinetic models (model 16 and model 27) for (a) LA and (b) EL, from the combination of Experimental Sets 1 and 2. The dashed lines represent the two-standard deviation limits.

The final rate expressions for the LHHW (model 16) and ER (model 27) mechanisms are shown in eqs 11 and 12. In Table 4, the relevant kinetic parameters are presented. The FD-generated experimental data of the combination of Experimental Sets 1 and 2, were compared to the predicted responses of models 16 and 27. The adequacy of the models proposed is demonstrated graphically in the parity plots of Figure 9, where the model predictions, both for the concentration of LA (Figure 9a) and for the concentration of EL (Figure 9b), fall around the interval of the maximum error, calculated as 2 times the experimental standard deviation. Although there are no obvious differences in the performance of models, as observed in Figure 9, a good agreement of both model predictions with the experimental data was established.
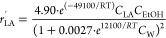
11
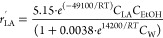
12

**Table 4:** Estimated Kinetic Parameters for the LHHW (Model 16) and ER (Model 27) Mechanisms and Their Respective 95% CI

	model 16	model 27
*A* (L^2^g^–1^s^–1^ mol^–1^)	4.90 (±2.14)	5.15 (±2.59)
*E*_a_ (J mol^–1^)	49,100 (±1300)	49,100 (±1500)
*K*_W,ref_ at 85 °C (L mol^–1^)	0.16 (±0.006)	0.45 (±0.03)
ΔΗ_W_ (J mol^–1^)	–12,100 (±2700)	–14,200 (±3700)

In this work, the apparent activation energy of the LA esterification with ethanol, *E*_a_, was 49.1 kJ/mol for both reaction kinetic models proposed, and the lumped kinetic rate constant, *k*, at the mean experimental temperature (reference temperature) of 85 °C was estimated equal to 3.056 × 10^–7^ L^2^g^–1^s^–1^ mol^–1^ for LHHW and 3.212 × 10^–7^ L^2^g^–1^s^–1^ mol^–1^ for ER, respectively. Table 5 summarizes the reported kinetics of existing studies on esterification systems using Amberlyst-15.

**Table 5:** Kinetic Parameters for Esterification Reactions from Different Studies

study	reaction system	catalyst size (μm)	acid to alcohol ratio	activation energy (*E*_a_) (kJ/mol)	kinetic rate constant (*k*) at 85 °C (L^2^ g^–1^ s^–1^ mol^–1^)	kinetic model
this work	levulinic acid + ethanol	800	1:9	49.1	3.056 × 10^–7^ (LHHW) 3.212 × 10^–7^ (ER)	LHHW, ER
Russo et al.^57^	levulinic acid + ethanol	300–600	1:1–1:5	85.3	7.381 × 10^–7^	pseudohomogeneous
Sharma et al.^86^	pentanoic acid +1-propanol	500	1:1 – 1:15	46.4	2.325 × 10^–7^	ER

Comparing with the existing literature, Russo et al.^57^ reported an apparent activation energy of 45.2 kJ/mol for Amberlyst-15 beads of 300 μm and 49.4 kJ/mol for beads of 600 μm, assuming that the LA esterification with ethanol was pseudohomogeneous first order reaction with respect to both reactants and expressing reaction rate in concentration terms. However, when the Thiele modulus was incorporated into the analysis, intrinsic kinetics were obtained, yielding an activation energy of 85.3 kJ/mol. Sharma et al.^86^ reported an activation energy of 46.4 kJ/mol for the esterification of pentanoic acid with 1-propanol in the presence of Amberlyst-15 500 μm particles, under conditions free of internal and external mass transfer resistances. The reaction followed ER kinetics, employing activity-based reaction rates.

Prior studies in the literature included adsorption experiments on Amberlyst-15 for other liquid-phase esterification reactions, with heterogeneous methyl acetate synthesis being an extensively examined reaction. Song et al.^87^ performed nonreactive binary adsorption experiments for the esterification of acetic acid with methanol at 45 °C and reported that water adsorption equilibrium constant is twice as high as this of methanol, followed by acetic acid and methyl acetate. Pöpken et al.^88^ compared the experimental data fitting of a pseudohomogeneous and a heterogeneous LHHW model. The adsorption experiments, conducted at 25 °C, revealed that the reaction components adsorb similarly on Amberlyst-15, as shown by the magnitude of their respective equilibrium adsorption constants. Based on the reaction rate expressions that fitted the experimental data in this work (eqs 11 and 12), water adsorption on the Amberlyst-15 surface is stronger than that of the rest of the reaction components, including ethanol that is the solvent. This is consistent with the above literature findings and aligns with the hypothesis that the polarity of the reaction components plays a pivotal role in the observed phenomena.^74^ Notably, water, being the most polar component, manifests the most pronounced inhibitory effect.

The enthalpy of adsorption for water in the LHHW proposed mechanism was estimated equal to Δ*Η*_W_ = −12.1 kJ/mol and Δ*Η*_W_ = −14.2 kJ/mol for the ER model, which is indicative of components adsorbed physically on the surface of the ion-exchange resin.^89^ At the reference temperature of 85 °C, the value of *K*_W_ (*K*_W,ref_) was calculated to be equal to 0.16 L/mol for the LHHW and 0.45 L/mol for the ER model. Comparing the estimated adsorption equilibrium constants with the ones provided by Sharma et al.,^66,86,90−92^ for an extensive range of liquid phase esterification reactions on Amberlyst-15, as shown in Table 6, the reported values for similar esterification systems are within the same order of magnitude as those obtained in this study. However, the enthalpies of adsorption for water are notably higher in Sharma et al.’s work, which can be attributed to the inclusion of nonideality of the reaction mixture in the reaction rate, differences in reaction media, and competitive adsorption effects.

**Table 6:** Comparison of the Enthalpy of Adsorption for Water, Δ*Η*_w_, and the Respective Adsorption Constant, *K*_w_, at Reference Temperature

authors	reaction system	Δ*Η*_w_ (kJ/mol)	*K*_w_ (L/mol) at *T*_ref_ = 85 °C
this work	levulinic acid + ethanol	–12.1 (LHHW)	0.16
		–14.2 (ER)	0.45
Sharma et al.^86^	pentanoic acid +1-propanol	–40.2	0.45
Sharma et al.^90^	nonanoic acid +1-propanol	–19.3	1.76
Sharma et al.^91^	pentanoic acid + methanol	–60.91	0.38 (*T* = 60 °C)
Sharma et al.^66^	nonanoic acid + ethanol	–46.5	1.01 (*T* = 75 °C)

From an engineering standpoint, the proposed kinetic models are valuable tools for reactor modeling and design due to their simplicity and practicality. However, two key aspects must be considered alongside internal mass transfer resistances when formulating intrinsic kinetics models: (i) the nonideality of the reaction mixture and (ii) phase partitioning due to absorption phenomena in the catalytic resin. Addressing the first aspect, rate equations can be expressed as functions of liquid-phase thermodynamic activities, calculated using models such as the nonrandom two-liquid^93^ or the universal quasichemical (UNIQUAC) model.^94^ Given the availability of Python libraries, such as Phasepy,^95^ integrating a thermodynamic model into the statistical analysis could refine model discrimination and parameter estimation, improving the predictive accuracy of kinetic descriptions, i.e., by computing activity coefficients along the reactor bed.

The second aspect—phase partitioning effects—has been explored for esterification reactions using the Flory–Huggins (FH) solution theory^96^ to determine phase equilibria between the bulk liquid and polymer phases.^97−100^ However, despite the complexity of the developed thermodynamic models, these studies have not demonstrated a clear improvement in kinetic modeling. In the present system, two observations suggest that further experimental investigation is warranted, stemming from the main differences between our work and previous studies. First, phase equilibrium studies in batch systems tend to be prolonged (in the time scale of hours to days) to guarantee that equilibrium is reached and estimate the parameters of the phase partition equilibrium for nonreactive mixtures, with the exception of Mazzotti et al.,^97^ who reported equilibrium within minutes for the synthesis of ethyl acetate. Second, Sainio et al.^99^ noted that in steady-state flow-through reactors (such as the SPSR used in this work), Amberlyst-15’s ability to influence phase equilibrium is significantly reduced. Moreover, depending on the operating conditions, the resin may enter the glass transition region, potentially affecting solvent sorption and, consequently, the reaction kinetics. Given that our reactor operates at 70–100 °C, it is crucial to determine whether the resin remains in a swollen state or approaches a transition region where its properties change.

## 5 Conclusions

The esterification of LA with ethanol in the presence of Amberlyst-15 was carried out in a continuous Single Peller String Reactor, as part of an automated platform controlled by LabVIEW. Due to the macroreticular structure of the catalyst and the commercially available sizes, internal mass transfer resistances could not be excluded, and therefore, the kinetic models reported reflect apparent kinetics. A systematic screening of kinetic models was performed, including model adequacy and estimation of identifiable parameters, of both simple and of increased complexity models, e.g., reaction rate expressions with high number of parameters. The combination of two experimental data sets (with and without the introduction of water in the feed), as well as the incorporation of temperature dependent equilibrium adsorption constants, sheds light on the effect of choosing the right design space and how fitting of seemingly inadequate models can be improved. Apparent kinetics and thermodynamic parameters were precisely estimated. A Langmuir–Hinshelwood-Hougen-Watson model and an Eley–Rideal model, including a temperature-dependent term for the adsorption equilibrium of water, were found to be the most appropriate kinetic models describing the production of EL from LA in the investigated design space. In both proposed models, it was observed that water exhibited a strong tendency to bind to the catalytic sites, thereby inhibiting the reaction. Due to the similarity between the two final reaction rate expressions, it was not possible to discriminate further. A more detailed exploration of phase equilibria is recommended to enhance the understanding of the interaction of the polymeric resin with the reaction components on the microscale and improve the predictive capability of the models.

## Supporting Information Available

Images of the SPSR, details of the LabVIEW user interface, NMR studies for the investigation of byproducts, DoE experimental data, esterification reaction equilibrium calculations, external and internal mass transfer study, heat transfer evaluation, and model fitting statistics including chi-square values from the automated kinetic experiments (PDF)
