# Supplementary material for: Esterification of Levulinic Acid to Ethyl Levulinate over Amberlyst-15 in Flow: Systematic Kinetic Model Discrimination and Parameter Estimation
Source: Ind Eng Chem Res. 2025 Apr 14;64(16):8064–78. doi: 10.1021/acs.iecr.4c04540 (PMC12022979; doi:10.1021/acs.iecr.4c04540)
Supplement: Supplementary file 1 — ie4c04540_si_001.pdf [file ie4c04540_si_001.pdf]

Supporting Information

Esterification of Levulinic Acid to Ethyl Levulinate over Amberlyst-15 in Flow: Systematic Kinetic Model Discrimination and Parameter Estimation

Eleni Grammenou[a], Maerthe Theresa Tillmann[a,b], Solomon Gajere Bawa[a], Arun Pankajakshan[a], Federico Galvanin[a] and Asterios Gavriilidis[a]

[a] Department of Chemical Engineering, University College London, Torrington Place, London, WC1E 7JE, U. K.

[b] Faculty of Mechanical Engineering, RWTH Aachen University, Elifschornsteinstraße 18, 52062 Aachen, Germany

Contents

S1. Images of the single pellet string reactor 2

S2. LabVIEW user interface 3

S3. NMR spectroscopy analysis 4

S4. Experimental results from the factorial experimental design 6

S5. Esterification reaction equilibrium calculations 9

S6. Evaluation of mass transport resistances 10

S6.1. External mass transfer resistances 10

S6.2. Internal mass transfer resistances 12

S7. Heat transfer evaluation 14

S8. Model fitting statistics 17

S8.1. Estimation of measurement error 17

S8.2. Parameter estimation and LoF 18

References 20

S1. Images of the single pellet string reactor

An image of a section of the single pellet string reactor is shown in Figure S1, taken using a digital optical microscope (VHX-600 Digital Microscope, Keyence). The images were captured when the particles were dry. During experimentation, the particles swelled by approximately 6%, as also reported by Waldron et al.,[1] however they had to be measured outside of the reactor at the end of the experimental cycle, as the light refraction through the reacting media caused a distortion of shape, as it can be seen from Figure S2.

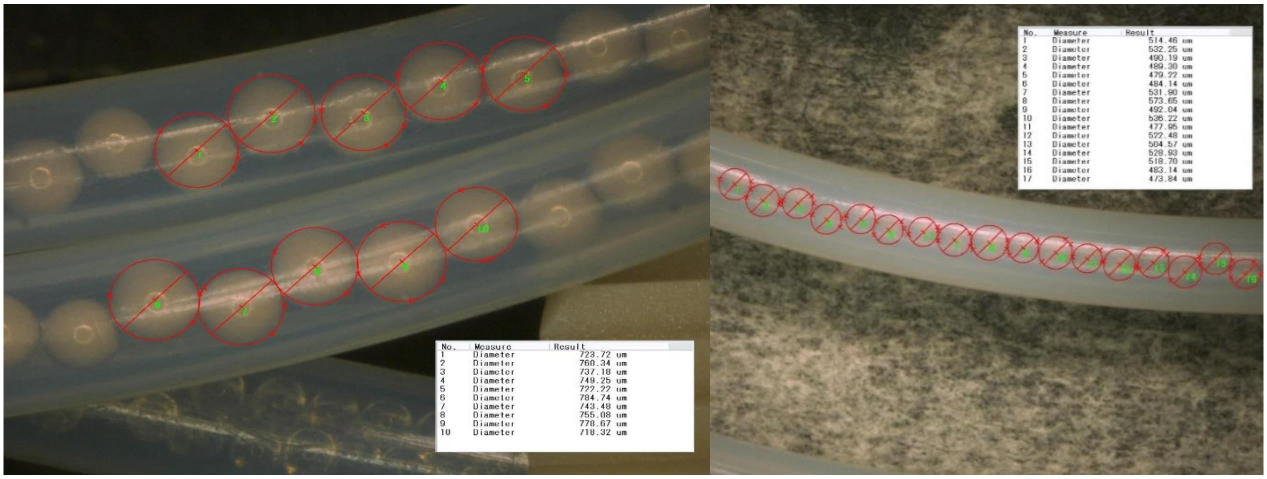

Figure S1. Microscope images of the dry Amberlyst-15 particles inside the reactor (a) from the 710-850 µm sieve fraction and (b) from the 500-600 µm sieve fraction.

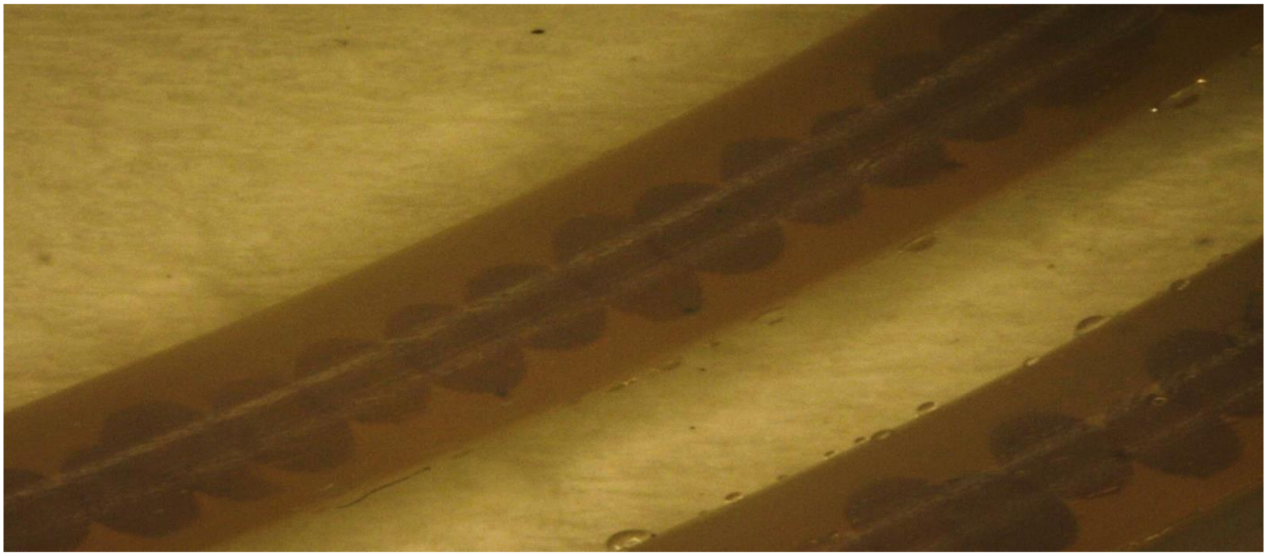

Figure S2. Microscope image of the wet Amberlyst-15 particles inside the reactor.

S2. LabVIEW user interface

The Laboratory Virtual Instrument Engineering Workbench (LabVIEW) environment was used for automating the experimental process. The LabVIEW code developed in this work allowed the user to run a list of pre-planned experiments, programmed in the LabVIEW user interface, as it can be seen from Figure S3. The actual values of the experimental conditions were displayed, as well as the concentrations for levulinic acid (LA) and ethyl levulinate (EL) at the end of each run, based on the area of the respective chromatogram peaks and their calibration curves. Temperature and pressure were monitored both by displaying their real-time values and their evolution through real-time running graphs.

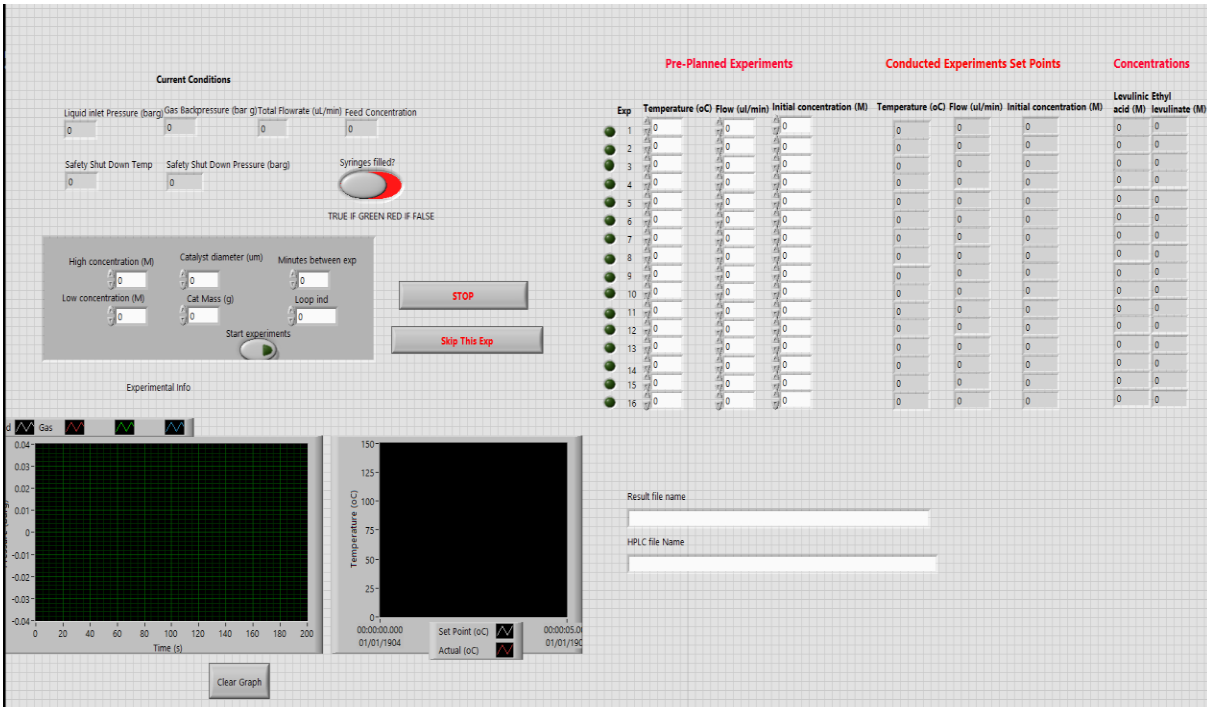

Figure S3. Screenshot of the LabVIEW user interface.

S3. NMR spectroscopy analysis

Nuclear Magnetic Resonance (NMR) spectroscopy was used to guarantee that no side-reactions were taking place under the experimental conditions investigated. For this reason, a sample was collected in the waste vessel, after the experiment ran for more than an hour to ensure that steady state conditions were achieved. A mixture of a similar composition was created using reactants and products purchased from the suppliers. Analysis was conducted with D_2O as the solvent. The spectra of ^1H NMR analysis are shown in Figure S4, whereas the spectra of ^{13}C NMR analysis are presented in Figure S5. Although, the intensity of the peaks is different due to the slightly different concentrations of the mixtures, it is important to note that the study focused on the qualitative analysis of the samples. The only differences between the reaction mixture (denoted by “a”) and the prepared solution (denoted by “b”) can be observed for the ^1H NMR spectrum at a shift of +2.04 ppm (Figure S4a), and for the ^{13}C NMR spectrum at +1.36 ppm and +119.46 ppm (Figure S5a).

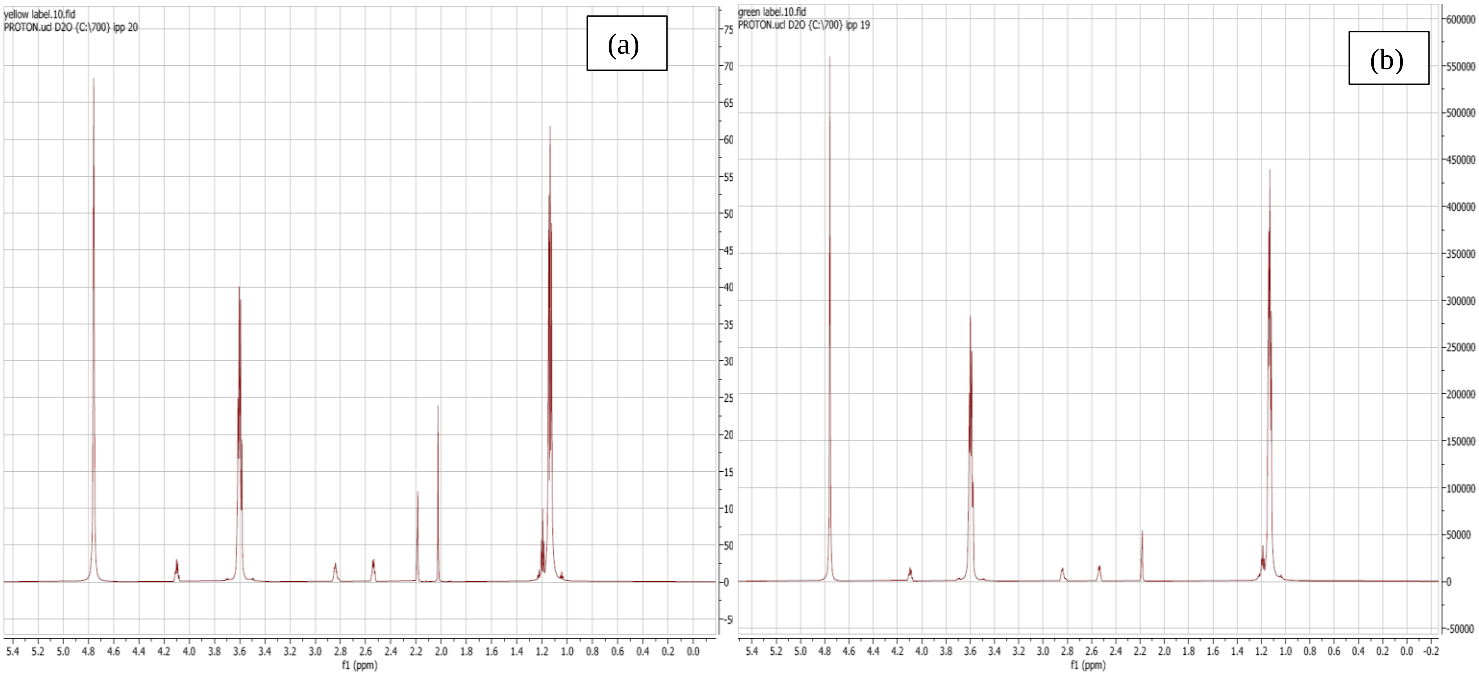

Figure S4. ^1H NMR spectra of (a) the effluent of the reactor and (b) pre-made solution of similar concentration.

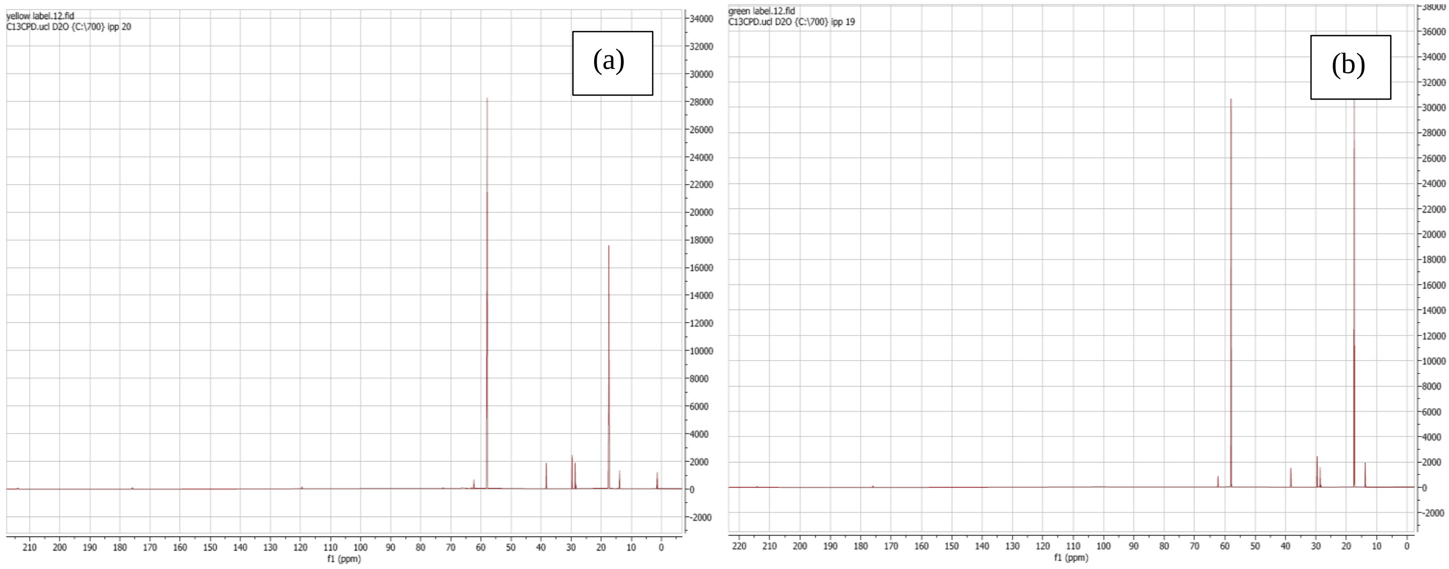

Figure S5. ^{13}C NMR spectra of (a) the effluent of the reactor and (b) pre-made solution of similar concentration.

Based on the NMR chemical shifts of impurities[2], the shift of the peaks can be attributed to the proton of CH_3 (for ^1H NMR) and the carbon of CN and CH_3 (for ^{13}C NMR) of acetonitrile. Acetonitrile cannot be formed as a by-product of the reaction, based on the possible reaction pathways.[3-5] Given that a mixture of acetonitrile and water were used as mobile phase for the HPLC analysis, traces of it potentially entered the system (after the exit of the reactor) in the sample loop of the HPLC sampler and dilutor and got collected in the waste vessel.

S4. Experimental results from the factorial experimental design

The experimental results obtained from the factorial experimental design are presented in Tables S1-4 for both Experimental Sets 1 and 2.

Table S1. LA measured concentration at the reactor outlet from three campaigns, each campaign was executed on a separate day and consisted of eight experiments. Data produced without addition of water in the feed solution (Experimental Set 1).

Experiment	Temperature (° C)	Flowrate (µl/min)	Inlet LA concentration (M)	Outlet LA concentration (M)			Average outlet LA concentration (M)
				Campaign			
				1	2	3	
1	70	20	0.8	0.377	0.401	0.389	0.389
2	70	20	1.6	0.870	0.910	0.894	0.891
3	70	60	0.8	0.631	0.624	0.610	0.622
4	70	60	1.6	1.315	1.305	1.302	1.307
5	100	20	0.8	0.059	0.066	0.073	0.066
6	100	20	1.6	0.192	0.208	0.229	0.210
7	100	60	0.8	0.301	0.305	0.311	0.306
8	100	60	1.6	0.695	0.710	0.701	0.702

Table S2. EL measured concentration at the reactor outlet from three campaigns, each campaign was executed on a separate day and consisted of eight experiments, as given in Table S1. Data produced without addition of water in the feed solution (Experimental Set 1).

Experiment	Outlet EL concentration (M)			Average outlet EL concentration (M)
	Campaign			
	1	2	3	
1	0.425	0.418	0.415	0.419
2	0.741	0.695	0.704	0.714
3	0.193	0.187	0.187	0.189
4	0.332	0.312	0.319	0.321
5	0.746	0.743	0.728	0.739
6	1.390	1.407	1.350	1.382
7	0.499	0.492	0.475	0.489
8	0.895	0.876	0.883	0.885

Table S3. LA measured concentration at the reactor outlet from three campaigns, each campaign was executed on a separate day and consisted of eight experiments. Data produced with the addition of water in the feed solution (Experimental Set 2).

Experiment	Temperature (° C)	Flowrate (µl/min)	Inlet LA concentration (M)	Inlet H_2O concentration (M)	Outlet LA concentration (M)			Average outlet LA concentration (M)
					Campaign			
					1	2	3	
1	70	20	0.8	4.18	0.637	0.658	0.632	0.642
2	70	20	1.6	4.23	1.274	1.312	1.261	1.293
3	70	60	0.8	4.18	0.745	0.763	0.760	0.756
4	70	60	1.6	4.23	1.522	1.517	1.553	1.531
5	100	20	0.8	4.18	0.245	0.288	0.261	0.266
6	100	20	1.6	4.23	0.595	0.614	0.615	0.608
7	100	60	0.8	4.18	0.518	0.557	0.532	0.537
8	100	60	1.6	4.23	1.090	1.093	1.125	1.103

Table S4. EL measured concentration at the reactor outlet from three campaigns, each campaign was executed on a separate day and consisted of eight experiments, as given in Table S3. Data produced with the addition of water in the feed solution (Experimental Set 2).

Experiment	Outlet EL concentration (M)			Average outlet EL concentration (M)
	Campaign			
	1	2	3	
1	0.165	0.153	0.144	0.154
2	0.284	0.293	0.265	0.281
3	0.055	0.048	0.063	0.055
4	0.109	0.101	0.107	0.106
5	0.490	0.519	0.501	0.505
6	0.930	0.917	0.919	0.922
7	0.266	0.252	0.258	0.259
8	0.479	0.508	0.466	0.485

S5. Esterification reaction equilibrium calculations

Prior to the introduction of water in the feed solution for Experimental Set 2, a preliminary estimation of whether the reaction could be considered reversible given the inlet concentrations of the components was conducted. Based on the kinetics reported by Russo et al.[6] for the same reaction in batch, the equilibrium constant was expressed by Equation S1.

$$K_{eq} = 3.18e^{\frac{-15140}{8.314}\left(\frac{1}{T}-\frac{1}{333}\right)} \qquad \text{(S1)}$$

where, T is the temperature (K).

Therefore, for the worst-case scenario, which is the one of higher temperatures (100 °C in this work), the equilibrium constant K_{eq} can be calculated equal to 5.71.

The ratio of outlet concentration of products over reactants, R, can be expressed as follows

$$R = \frac{C_W\, C_{EL}}{C_{LA} C_{EtOH}} \qquad \text{(S2)}$$

Substituting the values of experimental conditions 5-8 of Experimental Set 2 of Table S6 and S7 in Equation S2, the values of R are reported in Table S5. The higher R was found for experimental condition 5 equal to 0.606.

Table S5. Outlet concentrations of reactants (LA and EtOH) and products (EL and H_2O) and calculated ratio of outlet concentration of products over reactants, R for experimental conditions 5-8 of Experimental Set 2.

Experiment	Outlet LA concentration (M)	Outlet EL concentration (M)	Outlet H_2O concentration (M)	Outlet EtOH concentration (M)	R
5	0.266	0.505	4.446	13.932	0.606
6	0.608	0.922	4.838	12.097	0.606
7	0.537	0.259	4.717	14.178	0.160
8	1.103	0.485	5.333	12.534	0.187

Hence, since $R \ll K_{eq}$ for all cases investigated, it can be safely assumed that the conditions studied were far away from equilibrium.

S6. Evaluation of mass transport resistances

S6.1. External mass transfer resistances

The external mass transfer resistances can be assessed using the Mear's criterion[7]

$$\frac{-r'_{LA}\rho_b R_p n}{k_{LS}C_{LA,bulk}} < 0.15 \tag{S3}$$

where r'_{LA} is the observed rate of reaction of levulinic acid (kmol s^{-1} kg^{-1}), ρ_b is the bulk density of catalyst (kg m^{-3}), R_p is the radius of the catalyst particle (m), n is the reaction order, k_{LS} is the liquid-solid mass transfer coefficient (m s^{-1}) and $C_{LA,bulk}$ is the concentration of levulinic acid in the bulk solution (kmol m^{-3}), calculated as an average between inlet and outlet measured concentrations.

Using the high temperature experimental data shown in Tables S1 and S2 (experimental conditions 5-8 of Experimental Set 1), the rate of reaction is approximated as the average rate of reaction along the length of the reactor using

$$-r'_{LA} = \frac{Q\left(C_{LA,in} - C_{LA,out}\right)}{m_{A15}} \tag{S4}$$

where Q is the volumetric flowrate (L s^{-1}), $C_{LA,in}$ and $C_{LA,out}$ are the concentrations of levulinic acid at the inlet and outlet (mol L^{-1}) respectively and m_{A15} is the mass of Amberlyst-15 (g). The observed rate of reaction for the conditions studied was found to be <$8.98\cdot10^{-6}$ kmol s^{-1} kg^{-1}, as shown in Table S6.

Table S6. Observed rate of reaction for experiments 5-8 of Experimental Set 1.

Experiment	r'_{LA} (kmol s^{-1} kg^{-1})
5	2.42E-06
6	4.57E-06
7	4.88E-06
8	8.98E-06

The bulk catalyst density was calculated according to Equation S5

$$\rho_b = \rho_c(1 - \Phi_{rxr}) \tag{S5}$$

where ρ_c is the pellet catalyst density, and Φ_{rxr} is the void fraction of the reactor.

According to the manufacturer specifications[8], the Amberlyst-15 density is 610 kg m^{-3} and the bed void fraction was calculated to be 0.535 for a reactor of 1000 µm diameter packed with 800 µm spheres[9, 10]. The bulk catalyst density in the reactor was then calculated to be

$$\rho_b = 610(1 - 0.535) = 283.6 \text{ kg m}^{-3}$$

The solid-to-liquid mass transfer coefficient for Single Pellet String Reactors, was calculated based on the correlation of Templis and Papayannakos[11] for a single pellet string reactor with aspect ratio of 1.33 (consisting of a 2 mm I.D. tube and cylindrical particles of 1.5 mm) as an approximation.

$$Sh = 2.06 + 0.381Re^{0.614}Sc^{0.33} \tag{S6}$$

$$Re = \frac{\rho u d_p}{\mu} \quad \text{(S7)}$$

$$Sc = \frac{\nu}{D_M} \quad \text{(S8)}$$

$$Sh = \frac{k_{LS} d_p}{D_M} \quad \text{(S9)}$$

where ρ, μ and ν are the density (kg m^{-3}), dynamic viscosity (kg m^{-1} s^{-1}), kinematic viscosity (m^2 s^{-1}) of the levulinic acid solution, u is the superficial velocity (m s^{-1}), d_p is the particle diameter and D_M is the molecular diffusivity of levulinic acid in ethanol (m^2 s^{-1}).

The Wilke-Chang equation, Equation S10, was used to estimate the value of molecular diffusivity in cm^2 s^{-1}

$$D_M = \frac{7.4 * 10^{-8} (\varphi M_2)^{0.5} T}{\mu_2 V_1^{0.6}} \quad \text{(S10)}$$

where subscripts 1 and 2 are for the solute and solvent respectively, φ is the association parameter of the solvent, which is 1.5 for alcohols, M is the molecular weight (g mol^{-1}), V is the molar volume (ml mol^{-1}) and T is the temperature (K). For levulinic acid in ethanol the molecular weight of solvent is 46.07 g mol^{-1}, the molar volume of levulinic acid is 129.37 mL mol^{-1}. [12]

For the calculation of dynamic viscosity, we considered that the reacting mixture has the properties of the solvent, ethanol, and therefore, the Vogel equation was used, where T is temperature in Kelvin and with parameters taken from the Dortmund Data Bank.[13]

$$\mu = \exp\left(-7.3714 + \frac{2770}{74.6787 + T}\right) \quad \text{(S11)}$$

The ethanol liquid density was calculated based on the DIPPR105 equation with parameters taken by Dortmund Data Bank, based on the work of Golubev et al.[14]

$$\rho = \frac{99.3974}{0.310729^{1+(1-\frac{T}{513.18})^{0.305143}}} \quad \text{(S12)}$$

The values of the dimensionless numbers for the conditions studied, *Sc*, *Re* and *Sh*, as well as the k_{LS} and value of the Mear's criterion (assuming a first order reaction) are shown in Table S7.

Table S7. Calculated values of dimensionless numbers and Mear's criterion parameter.

Experiment	*Sc*	*Re*	*Sh*	k_{LS} (m^2/s)	Mear's criterion parameter (-)
5	109.22	0.78	3.62	1.80E-05	0.019
6	109.22	0.78	3.62	1.80E-05	0.018
7	109.22	2.34	5.12	2.55E-05	0.027
8	109.22	2.34	5.12	2.55E-05	0.025

The dimensionless parameter of Mear's criterion was calculated to be significantly less than 0.15, hence suggesting that external mass transfer can be ignored.

S6.2. Internal mass transfer resistances

The Weisz-Prater criterion is used for the assessment of internal mass transfer resistances.[7]

$$C_{WP} = \frac{-r'_{LA}\rho_c R_p{}^2}{D_e C_{LA,s}} \tag{S13}$$

where ρ_c is the catalyst particle density, $C_{LA,s}$ is the concentration of levulinic acid at the catalyst surface and D_e is the effective diffusivity of levulinic acid in the catalyst particle (m^2/s), which is calculated according to the following expression

$$D_e = \frac{D_M \Phi_p \sigma_c}{\tau} \tag{S14}$$

where, Φ_p is the catalyst particle porosity, σ_c is the constriction factor and τ is the catalyst tortuosity, and the molecular diffusivity of levulinic acid in the ethanol solvent, D_M is calculated based on Equation S10. Since the external mass transfer resistances can be neglected, the concentration of levulinic acid at the catalyst surface, $C_{LA,s}$ can be approximated with the concentration of levulinic acid at the bulk solution, $C_{LA,bulk}$. The catalyst pellet porosity Φ_p was taken equal to 0.32, the constriction factor σ_c value equal to 0.8 and the tortuosity τ was taken equal to 3.[15] Replacing the values into the Equation S13 and S14, the Weisz-Prater parameter was then calculated for all experimental conditions of Experimental Set 1 (without water) as shown in Table S8.

Table S8. Weisz-Prater criterion, C_{WP} for the estimation of internal mass transfer resistances in Amberlyst-15 for the conditions of Experimental Set 1.

Experiment	Temperature (° C)	Flowrate (μl/min)	Inlet LA concentration (M)	Observed reaction rate (kmol kg^{-1} s^{-1})	C_{WP}
1	70	20	0.8	1.37E-06	0.74
2	70	20	1.6	2.36E-06	0.64
3	70	60	0.8	1.78E-06	1.03
4	70	60	1.6	2.92E-06	0.80
5	100	20	0.8	2.42E-06	0.77
6	100	20	1.6	4.57E-06	0.73
7	100	60	0.8	4.88E-06	1.55
8	100	60	1.6	8.98E-06	1.43

Since the C_{WP} value was calculated >1 for some of the experimental conditions, there was indication of internal mass transfer resistances. The calculation of Weisz-Prater criterion was based on the assumption of a first order reaction.

In more complicated kinetic models, such as LHHW and ER, the calculation of the internal mass transfer resistances involves the numerical solution of the differential mass and energy balances in the catalyst porous particle[16], which requires the knowledge of the kinetic parameters a priori. Since the purpose of this work was to estimate the esterification reaction kinetic parameters, this would require a

trial-and-error approach, which would increase complexity and be extremely time consuming. Thus, we did not take internal mass transfer resistances into account, and hence the kinetics obtained are categorised as apparent kinetics. An experimental investigation of internal mass transfer resistances was also conducted by testing different average particle sizes (755 μm and 550 μm, which due to swelling resulted in average diameters of 800 μm and 580 μm). A partial factorial design of experiments (4 experiments of those reported in Table S8) was conducted and repeated twice for different reactors loaded with the same amount of catalyst. As shown in Figure S6, an average difference in conversion of 8% was observed for the experimental conditions studied between the bigger and smaller particles, suggesting the presence of internal mass transfer limitations.

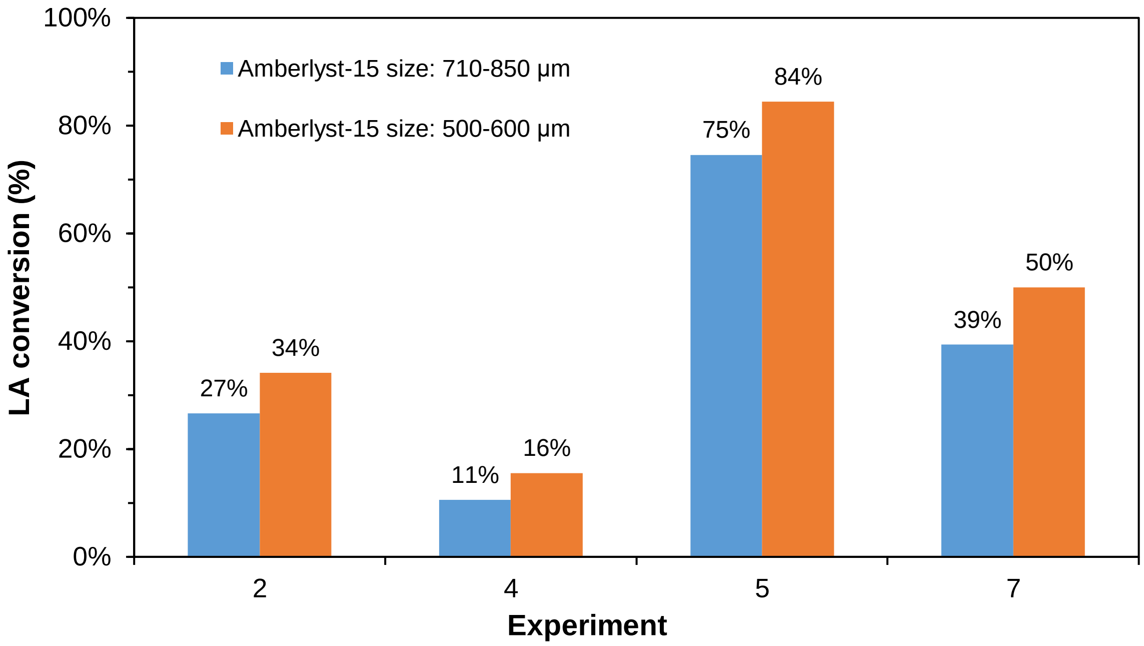

Figure S6. Conversion of LA using different Amberlyst-15 catalyst sizes. Each experiment corresponds to the experimental condition with the same number displayed in Table S8.

S7. Heat transfer evaluation

To ensure that the reaction mixture reaches the desired temperature, the preheating section must be of sufficient length to allow the fluid to attain the target temperature. The power required, $\dot{Q}$ (J s^{-1}), to increase the reaction fluid temperature from room temperature, T_{room} (K) equal to 293.15 K, to the glycerol bath temperature, T_{bath} (K) equal to 373.15 K, is given by [17]

$$\dot{Q} = \dot{m} c_{p,EtOH} (T_{bath} - T_{room}) \tag{S15}$$

where $c_{p,EtOH}$ (J kg^{-1} K^{-1}) is the specific heat capacity of ethanol[18] (2570 J kg^{-1} K^{-1}) and $\dot{m}$ (kg s^{-1}) is the mass flowrate of reaction fluid (assumed to be pure ethanol). Under the most demanding conditions - heating ethanol from 25 °C to 100 °C at a maximum flowrate of 60 µL/min), the power required, calculated from Equation S15, was 0.15 W. The heat exchange area of the empty tube before the fluid reaches the catalyst particles, A (m^2) required to achieve this temperature difference is calculated by

$$\dot{Q} = UA\Delta T_{LM} \tag{S16}$$

where U is the overall heat transfer coefficient (W m^{-2} K^{-1}) and ΔT_{LM} is the log mean temperature difference (K) [17, 19]. The bath temperature cannot be directly used as the outlet temperature, as this would require an infinitely large heat transfer area. Instead, a slightly lower value (0.5 °C below the bath temperature) was used. The log mean temperature difference was calculated using Equation S17, to be 15.0 °C.

$$\Delta T_{LM} = \frac{(T_{bath} - T_{in}) - (T_{bath} - T_{out})}{ln\left(\frac{T_{bath} - T_{in}}{T_{bath} - T_{out}}\right)} \tag{S17}$$

U was calculated from [17, 19]

$$\frac{1}{U} = \frac{1}{h_i} + \frac{d_i}{2k_t} ln\left(\frac{d_o}{d_i}\right) + \frac{d_i}{d_o h_o} \tag{S18}$$

where, h_i is the heat transfer coefficient (W m^{-2} K^{-1}) inside the tube, d_i and d_o are the inner and outer diameter (m) of the reactor tubing (equal to 1 mm and 1.59 mm respectively), k_t is the thermal conductivity (W m^{-1} K^{-1}) of the reactor tubing (which for PEEK is 0.25 W m^{-1} K^{-1}) and h_o is the heat transfer coefficient (W m^{-2} K^{-1}) outside the tube. The internal heat transfer coefficient was obtained from the Nusselt number for laminar flow in tubes for a constant wall temperature [17, 19].

$$Nu = \frac{h_i d_i}{k_{EtOH}} = 3.66 \tag{S19}$$

The internal heat transfer coefficient, h_i, was determined to be 612 W m^{-2} K^{-1}. The thermal conductivity of ethanol used in these calculations was 0.167 W m^{-1} K^{-1}. The external heat transfer coefficient, h_o depends on the properties of the glycerol filled agitated oil bath. The correlation for an agitated tank using a Rushton turbine was used (Equation S20), as an approximation[20]. The change in viscosity between the bulk glycerol and the glycerol close to the walls was considered negligible.

$$\frac{h_o d_T}{k_{glycerol}} = 0.81\left(\frac{\rho_{glycerol} N d_{imp}^2}{\mu_{glycerol}}\right)^{0.64} \left(\frac{c_{p,glycerol}\mu_{glycerol}}{k_{glycerol}}\right)^{0.33} \tag{S20}$$

In Equation S20, N is the number of rotations per second (known to be 10 from the hot plate stirrer set point), d_{imp} is the diameter of the impeller (m), d_T is the diameter of the tank (m), $\rho_{glycerol}$ is the density of glycerol (kg m^{-3}), $c_{p,glycerol}$ is the specific heat capacity of glycerol (J kg^{-1} K^{-1}), $\mu_{glycerol}$ is the viscosity of glycerol (Pa s) and $k_{glycerol}$ is its thermal conductivity (W m^{-1} K).

In our system, the magnetic stir bar diameter was 0.03 m and the diameter of the tank used was 0.1 m. The physical properties of glycerol were taken from the literature [21, 22]: at 100 °C glycerol has a density of 1187 kg m^{-3}, a viscosity of 0.0046 Pa s, a specific heat capacity of 2744.5 J kg^{-1} K^{-1} and a thermal conductivity of 0.288 W m^{-1} K^{-1}.[23] Using these parameters, h_o was estimated to be 1171 W m^{-2} K^{-1}. By combining the thermal conductivity of the reactor wall with the internal and external heat transfer coefficients (Equation S18), the overall heat transfer coefficient, U was found to be 324.7 W m^{-2} K^{-1}. Using Equation S16, the area was calculated to be 0.32 cm^2 and hence the length of tubing required for heating was calculated to be 1 cm. Thus, since the preheating section submerged in the reactor exceeded this length (the empty tube for feed preheating was 2 cm) and furthermore there was a 5 cm long section with glass beads placed upstream of the catalytic bed, it is expected that the reaction mixture reached the bath temperature before entering the catalytic bed.

Additionally, a calculation was performed to find the temperature increase along the catalytic bed. We considered the most severe case, that is the experiment with conversion, X = 84%, $C_{LA,0}$ = 1.6 M (and $C_{EtOH,0}$ = 14.32 M), flowrate of 20 µL/min and a reaction temperature of 100 °C. For a tubular pseudohomogeneous reactor incorporating a heating jacket, Equation S21 can be used[7].

$$\frac{dT}{dm_{A15}} = \frac{r'_{LA}\,(-\Delta H_R(T)) - \frac{U\alpha}{\rho_b}\,(T - T_\alpha)}{\sum F_i c_{p,i}} \qquad \text{(S21)}$$

where, r'_{LA} is the rate of reaction of levulinic acid per mass of catalyst (mol g^{-1} s^{-1}) as calculated by the proposed model mechanisms of Equation 12 (main paper), $\Delta H_R(T)$ is the heat of reaction at temperature T (J mol^{-1}), F_i is the molar flowrate of component i (mol s^{-1}) calculated based on reaction stoichiometry and inlet conditions, $c_{p,i}$ is heat capacity of component i (J mol^{-1} K^{-1}), U is overall heat-transfer coefficient, taken for the worst case scenario of an empty tube in laminar flow as 324.7 W m^{-2} K^{-1} (as calculated above), α is the heat exchange area per volume of reactor (m^{-1}) equal to 3200 m^{-1}, ρ_b is the catalyst bulk density equal to 283.6 kg m^{-3} and T_α is the temperature of the glycerol bath at 100 °C. The specific heat capacities were assumed to be constant over the temperature range of 25 °C to the final temperature. The enthalpy of reaction was calculated by:

$$\Delta H_R(T) = (H^0_{f,W} + H^0_{f,EL} - H^0_{f,EtOH} - H^0_{f,LA}) + \Delta c_p\,(T - T_R) \qquad \text{(S22)}$$

where, Δc_p accounts for the change in heat capacity due to the reaction and T_R is a reference temperature (25 °C). The thermodynamic properties of the components can be found in Table S9.

Table S9. Specific heat capacities and enthalpies of formation at standard conditions for the reaction components.

Component	$c_p\ (\frac{\mathrm{J}}{\mathrm{mol\ K}})$ [24, 25]	$H_f{}^0 (\frac{\mathrm{kJ}}{\mathrm{mol}})$ [26]
LA	268.1	-677.9
EtOH	118.4	-273.9
EL	317.5	-679.0
W	75.6	-284.0

Integrating simultaneously the mole balance for LA (Equation 1, main paper) along with the mole balances of the other components (obtained through reaction stoichiometry, not shown) and the energy balance (Equation S21), from 0 to 0.1 g of catalyst mass and for T_{inlet} = 100 °C, the temperature at the reactor outlet was found to be T_{outlet} = 100.1 °C and there was no formation of hot spots within the bed. Therefore, the reactor can be safely assumed to operate under nearly isothermal conditions. The conversion and temperature profile along the length of the reactor can be found in Figure S7.

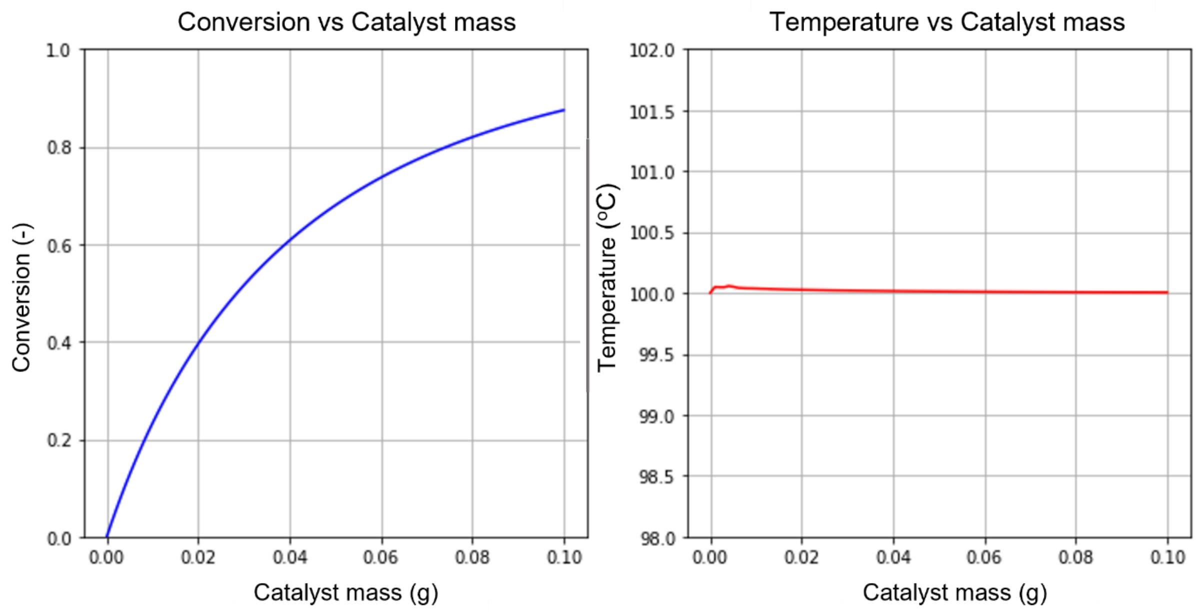

Figure S7. Conversion (left) of levulinic acid and temperature (right) as a function of catalyst mass for $C_{LA,0}$ = 1.6 M, $C_{EtOH,0}$ = 14.32 M, flowrate = 20 µL/min, inlet feed temperature = 100 °C.

S8. Model fitting statistics

A general reaction system model, comprising the reactor model as well as the kinetic model is represented by a set of differential and algebraic equations (DAEs), the solution of which characterises the modelled system behaviour.

$$\boldsymbol{f}(\dot{\boldsymbol{x}}(t), \boldsymbol{x}(t), \boldsymbol{u}(t), \boldsymbol{\theta}) = 0 \qquad \text{(S23)}$$
$$\hat{\boldsymbol{y}} = \boldsymbol{g}(\boldsymbol{x}(t), \boldsymbol{u}(t), \boldsymbol{\theta})$$

In Equation S23, $\boldsymbol{x}$ is the N_x dimensional vector of the state variables, $\dot{\boldsymbol{x}}$ is the vector of first derivatives of the state variables, $\boldsymbol{u}$ is the N_u dimensional vector of inputs or control variables, defined by the experimental conditions, $\boldsymbol{\theta}$ is the N_θ dimensional vector of the kinetic model parameters and $\hat{\boldsymbol{y}}$ is the $N_{\hat{y}}$ vector of model predictions of the outputs or measurable responses, $\boldsymbol{y}$. The vector of functions representing the state equation is denoted as $\boldsymbol{f}$, whereas the vector of functions representing the output equation is described by $\boldsymbol{g}$, both being usually nonlinear functions of $\boldsymbol{\theta}$ and $\boldsymbol{u}$ for chemical reaction systems.[27]

In this study, $\boldsymbol{x}$ comprises the concentrations of levulinic acid (C_{LA}), ethanol (C_{EtOH}), ethyl levulinate (C_{EL}) and water (C_W), $\boldsymbol{u}$ comprises the reaction temperature, the feed concentration and the total flowrate, $\boldsymbol{\theta}$ includes model parameters, namely KP_1 and KP_2 (i.e., re-parametrised pre-exponential factor (A) and energy of activation (E_α) (Equations 3-5 of the main paper)) and constant values of K_i (for the case of temperature-independent models) or $KP_{ads_{1,i}}$ and $KP_{ads_{2,i}}$ (when temperature dependence is considered) as re-parametrised values for the enthalpy of adsorption of the i different species (Equations 6-8).

Data collected from different experimental conditions are represented by the experimental design vector $\boldsymbol{\varphi}$, as shown in Equation S24.

$$\boldsymbol{\varphi} = [\boldsymbol{x}^0, \boldsymbol{u}, m_{A15}] \qquad \text{(S24)}$$

In Equation S24, $\boldsymbol{x}^0$ is the initial state vector consisting of the experimental conditions and m_{A15} is the mass of Aberlyst-15. The design space $\boldsymbol{D}$ includes all possible variants of experimental conditions and it is bounded by physical constraints of the experimental set up. For data collected by executing N_{exp} experiments and measuring N_y responses at steady state, the measurement response matrix $\boldsymbol{y}$ with dimensions $N_{exp} \times N_y$ can be defined.

S8.1. Estimation of measurement error

To estimate the measurement error, it is assumed that the N_y measured responses (in this work, the measured responses are two, the concentration of LA (C_{LA}) and EL (C_{EL})) of a true model are corrupted with uncorrelated normally distributed measurement error with zero mean vector and standard deviation σ_j. Therefore, the measurement error is expressed as $N_y \times N_y$ dimensional covariance matrix $\boldsymbol{\Sigma}$ with σ_j^2 as entries on the diagonal. Furthermore, the error is attributed solely on the system response variables and not on systematic bias or error from other sources like imperfect input control and thus assumed to be constant over the entire design space of a dataset.

S8.2. Parameter estimation and LoF

Parameter estimation concerns the identification of values for the unknown parameters $\boldsymbol{\theta}$ in the kinetic expression. These values are the solution of an optimisation problem that involves the minimisation of lack of fit (LoF) expressed as a function of the residuals $\boldsymbol{\varepsilon}_i$.The vector of residuals $\boldsymbol{\varepsilon}_i$ is the difference between model prediction $\hat{\boldsymbol{y}}_i$ and the measured model response $\boldsymbol{y}_i$ for the i-th experiment for the N_y measurement variables (Equation S25). The LoF was assessed using the χ^2 value calculated as the weighted sum of squared of the residuals $\boldsymbol{\varepsilon}_i$ (Equation S26) obtained after maximum likelihood parameter estimation. The χ^2 value is a function of the kinetic parameters vector, $\boldsymbol{\theta}$, and is minimised at the optimal parameter estimates, $\hat{\boldsymbol{\theta}}$.

$$\boldsymbol{\varepsilon}_i = \boldsymbol{y}_i - \hat{\boldsymbol{y}}_i \quad \forall i = 1, \dots, N_y \tag{S25}$$

$$\chi^2 = \sum_{i=1}^{N_{exp}} \sum_{j=1}^{N_y} \boldsymbol{\Sigma}_{j,j}^{-1} \cdot \varepsilon_{i,j}{}^2 \tag{S26}$$

As parameter estimates are the product of model fitting to experimental data affected by a measurement error, $\boldsymbol{\Sigma}$, parameter estimates are uncertain. The uncertainty is quantified by the $N_\theta \times N_\theta$ dimensional variance-covariance matrix of parameter estimates, $\hat{\boldsymbol{V}}_{\boldsymbol{\theta}}$ that is a common measure of the parameter estimates' precision, defined as the inverse of the observed Fisher information matrix $\hat{\boldsymbol{H}}_{\boldsymbol{\theta}}$.[28]

$$\hat{\boldsymbol{V}}_{\boldsymbol{\theta}}(\hat{\boldsymbol{\theta}}) \cong [\hat{\boldsymbol{H}}_{\boldsymbol{\theta}}(\hat{\boldsymbol{\theta}})]^{-1} = \left[[\hat{\boldsymbol{V}}_{\boldsymbol{\theta}}^{\boldsymbol{0}}]^{-\boldsymbol{1}} + \sum_{i=1}^{N_{exp}} \hat{\boldsymbol{S}}^T (\boldsymbol{\varphi}_i, \hat{\boldsymbol{\theta}}) \, \boldsymbol{\Sigma}^{-1} \hat{\boldsymbol{S}}(\boldsymbol{\varphi}_i, \hat{\boldsymbol{\theta}}) \right]^{-1} \tag{S27}$$

In Equation S27, $\hat{\boldsymbol{V}}_{\boldsymbol{\theta}}^{\boldsymbol{0}}$ is the a-priori covariance matrix of the model parameters calculated from the potential prior knowledge of parametric space (in this study, it was a null matrix as there was no preliminary information provided), $\hat{\boldsymbol{S}}\,(\boldsymbol{\varphi}_i, \hat{\boldsymbol{\theta}})$ represents the linear approximation for the sensitivity of model predictions $\hat{\boldsymbol{y}}$ with respect to model parameters $\hat{\boldsymbol{\theta}}$. For experiment i, $\hat{\mathbf{S}}$ is defined as a $N_y \times N_\theta$ dimensional matrix as follows.

$$\hat{\boldsymbol{S}}\,(\boldsymbol{\varphi}_i, \hat{\boldsymbol{\theta}}) = \begin{pmatrix} \frac{\partial \hat{y}_{i,1}(\hat{\boldsymbol{\theta}})}{\partial \hat{\theta}_1} & \cdots & \frac{\partial \hat{y}_{i,1}(\hat{\boldsymbol{\theta}})}{\partial \hat{\theta}_{N_\theta}} \\ \vdots & \ddots & \vdots \\ \frac{\partial \hat{y}_{i,N_y}(\hat{\boldsymbol{\theta}})}{\partial \hat{\theta}_1} & \cdots & \frac{\partial \hat{y}_{i,N_y}(\hat{\boldsymbol{\theta}})}{\partial \hat{\theta}_{N_\theta}} \end{pmatrix} \forall\, \boldsymbol{i} = 1, \dots, N_{exp} \tag{S28}$$

Parameter correlation can be quantified from the parameter correlation matrix $\boldsymbol{C}$ for which the entry of row i and column j is calculated based on the entries of the variance-covariance matrix of parameter estimates $\hat{\boldsymbol{V}}_{\boldsymbol{\theta}}$, as shown in Equation S29.

$$C_{i,j} = \frac{\hat{V}_{\theta_{i,j}}}{\sqrt{\hat{V}_{\theta_{i,i}}}\sqrt{\hat{V}_{\theta_{j,j}}}} \quad \forall\, i,j = 1, \dots, N_\theta \tag{S29}$$

From the parameter variance-covariance matrix $\hat{\boldsymbol{V}}_{\boldsymbol{\theta}}$, the linear Confidence Interval (*CI*) for the i-th parameter $\hat{\theta}_i$ is computed as follows.[29]

$$CI_i = \sqrt{\hat{V}_{\theta_{i,i}}} \cdot t_2(p - value, DoF)\ \forall\ i = 1, \ldots, N_\theta \qquad \text{(S30)}$$

with $t_2(p - value, DoF)$ being the t-value of a two-tailed Student's t-distribution dependent on user defined significance level (here 5%) and the $N_{exp} \cdot N_y - N_\theta$ degrees of freedom (*DoF*) of the model.

The following tables contain the quantitative model fitting information in the form of the LoF metric, the chi-square value (χ^2) and its respective threshold value (χ^2_{ref}) from a χ^2 test with a 95% significance level and $N_{exp} \cdot N_y - N_\theta$ degrees of freedom. Data of Table S10 are presented graphically in Figures 5-7 of the main paper and the data of Table S11 are depicted in Figure 8 (a-c).

Table S10. Model fitting statistics based on the LoF calculation (χ^2 test) by means of χ^2 and χ^2_{ref} values for the experimental spaces explored in this work: Experimental Set 1 (without water in the feed), Experimental Set 2 (with water in the feed) and combination of Experimental Sets 1 and 2.

Model No.	Experimental Set 1		Experimental Set 2		Experimental Set 1 and 2 combined	
	χ^2	χ^2_{ref}	χ^2	χ^2_{ref}	χ^2	χ^2_{ref}
Model 1	192.8	62.8	176.7	60.5	6640.2	115.4
Model 2	3190.9	62.8	991.6	60.5	10636.6	115.4
Model 3	56.2	58.1	80.3	55.8	187.7	110.9
Model 4	56.2	59.3	102.7	56.9	220.9	112.0
Model 5	56.2	59.3	80.3	56.9	6408.8	112.0
Model 6	192.8	60.5	176.7	58.1	6640.2	113.1
Model 7	56.2	59.3	100.3	56.9	193.3	112.0
Model 8	56.2	60.5	109.4	58.1	220.9	113.1
Model 9	56.2	60.5	100.3	58.1	6408.8	113.1
Model 10	192.8	61.7	176.7	59.3	6640.2	114.3
Model 11	56.7	59.3	80.3	56.9	187.7	112.0
Model 12	56.7	60.5	102.9	58.1	220.9	113.1
Model 13	56.7	60.5	80.3	58.1	6411.2	113.1
Model 14	192.8	61.7	176.7	59.3	6640.2	114.3
Model 15	56.7	60.5	100.3	58.1	193.3	113.1
Model 16	56.7	61.7	109.4	59.3	220.9	114.3
Model 17	56.7	61.7	100.3	59.3	6411.2	114.3
Model 18	56.5	59.3	100.7	56.9	183.3	112.0
Model 19	56.5	60.5	124.3	58.1	201.1	113.1
Model 20	56.5	60.5	100.7	58.1	6409.0	113.1
Model 21	192.8	61.7	176.7	59.3	6640.2	114.3
Model 22	56.9	59.3	82.0	56.9	178.9	112.0
Model 23	56.9	60.5	124.3	58.1	201.1	113.1

Model 24	56.9	60.5	82.0	58.1	6411.3	113.1
Model 25	192.8	61.7	176.7	59.3	6640.2	114.3
Model 26	56.9	60.5	103.8	58.1	183.3	113.1
Model 27	56.9	61.7	124.4	59.3	201.1	114.3
Model 28	56.9	61.7	100.7	59.3	6411.3	114.3

Table S11. Model fitting statistics based on the LoF calculation (χ^2 test) by means of χ^2 and χ^2_{ref} values for the reformulated models with the minimum $\chi^2$of Table S10. The χ^2 and χ^2_{ref} values are calculated for the combined experimental sets on the hypothesis of a) Constant adsorption equilibrium parameters, b) Temperature dependency of the adsorption equilibrium parameters for water and EL and c) Temperature dependency of the adsorption equilibrium constant only for water.

Model No.	Constant adsorption equilibrium for water and EL		Temperature dependence of adsorption equilibrium for water and EL		Temperature dependence of adsorption equilibrium for water and constant adsorption equilibrium for EL	
	χ^2	χ^2_{ref}	χ^2	χ^2_{ref}	χ^2	χ^2_{ref}
Model 15	193.3	113.1	127.1	110.9	127.4	112
Model 16	220.9	114.3	144.2	113.1	144.2	113.1
Model 26	183.3	113.1	135.5	110.9	136.8	112
Model 27	201.1	114.3	142.5	113.1	142.5	113.1

References

1. Waldron, C.; Pankajakshan, A.; Quaglio, M.; Cao, E.; Galvanin, F.; Gavriilidis, A., An autonomous microreactor platform for the rapid identification of kinetic models. *React. Chem. Eng.* **2019,** 4, (9), 1623-1636.

2. Babij, N. R.; McCusker, E. O.; Whiteker, G. T.; Canturk, B.; Choy, N.; Creemer, L. C.; Amicis, C. V. D.; Hewlett, N. M.; Johnson, P. L.; Knobelsdorf, J. A.; Li, F.; Lorsbach, B. A.; Nugent, B. M.; Ryan, S. J.; Smith, M. R.; Yang, Q., NMR chemical shifts of trace impurities: Industrially preferred solvents used in process and green chemistry. *Organic Process Research & Development* **2016,** 20, (3), 661-667.

3. Su, F.; Ma, L.; Song, D.; Zhang, X.; Guo, Y., Design of a highly ordered mesoporous $H_3PW_{12}O_{40}/ZrO_2$–Si(Ph)Si hybrid catalyst for methyl levulinate synthesis. *Green Chem.* **2013,** 15, (4), 885.

4. Ramli, N. A. S.; Sivasubramaniam, D.; Amin, N. A. S., Esterification of levulinic acid using ZrO2-supported phosphotungstic acid catalyst for ethyl levulinate production. *Bioenerg. Res.* **2017,** 10, (4), 1105-1116.

5. Enumula, S. S.; Gurram, V. R. B.; Chada, R. R.; Burri, D. R.; Kamaraju, S. R. R., Clean synthesis of alkyl levulinates from levulinic acid over one pot synthesized WO_3-SBA-16 catalyst. *Journal of Molecular Catalysis A: Chemical* **2017,** 426, 30-38.

6. Russo, V.; Hrobar, V.; Mäki-Arvela, P.; Eränen, K.; Sandelin, F.; Di Serio, M.; Salmi, T., Kinetics and modelling of levulinic acid esterification in batch and continuous reactors. *Top Catal* **2018,** 61, (18-19), 1856-1865.

7. Fogler, H. S., *Elements of Chemical Reaction Engineering*. 5th ed.; Prentice Hall: Boston, 2016.

8. DuPont AmberLyst™ 15DRY polymeric catalyst. https://www.dupont.com/content/dam/water/amer/us/en/water/public/documents/en/IER-AmberLyst-15DRY-PDS-45-D00927-en.pdf

9. Dixon, A. G., Correlations for wall and particle shape effects on fixed bed bulk voidage. *Can. J. Chem. Eng.* **1988,** 66, (5), 705-708.

10. Dixon, A. G., Wall and particle-shape effects on heat transfer in packed beds. *Chemical Engineering Communications* **1988,** 71, (1), 217-237.

11. Templis, C. C.; Papayannakos, N. G., Liquid-to-particle mass transfer in a structured-bed minireactor. *Chem. Eng. Technol.* **2017,** 40, (2), 385-394.

12. Escobar, A. M.; Blanco, M. N.; Martínez, J. J.; Cubillos, J. A.; Romanelli, G. P.; Pizzio, L. R., Biomass derivative valorization using nano core-shell magnetic materials based on Keggin-heteropolyacids: Levulinic acid esterification kinetic study with n-Butanol. *Journal of Nanomaterials* **2019,** 2019, 1-14.

13. Dortmund Data Bank, Liquid dynamic viscosity-Calculation by Vogel equation. In http://ddbonline.ddbst.de/VogelCalculation/VogelCalculationCGI.exe, 2023.

14. Golubev, I. F.; Vasil'kovskaya, T. N.; Zolin, V. S., Experimental study of the density of aliphatic alcohols at various temperatures and pressures. *Journal of Engineering Physics* **1980,** 38, (4), 399-401.

15. Takagaki, A., Kinetic analysis of aqueous-phase cyclodehydration of 1,4-butanediol and erythritol over a layered niobium molybdate solid acid. *Catal. Sci. Technol.* **2016,** 6, (3), 791-799.

16. Olatunde, A. O.; Olafadehan, O. A.; Usman, M. A., Computation of effectiveness factor for methanol steam reforming over $Cu/ZnO/Al_2O_3$ catalyst pellet. *Appl Petrochem Res* **2020,** 10, (1), 35-47.

17. Sinnott, R. K.; Towler, G., *Chemical Engineering Design: SI Edition*. Elsevier: Oxford, 2009.

18. Domalski, E. S.; Hearing, E. D., Condensed phase heat capacity data. In *NIST Chemistry WebBook, NIST Standard Reference Database Number 69*, P.J. Linstrom; W.G. Mallard, Eds. National Institute of Standards and Technology: Gaithersburg MD, 20899, 2018.

19. Serth, R. W.; Lestina, T., *Process Heat Transfer : Principles and Applications, 2nd edn.* Academic Press: Oxford, 2014.

20. Rosa, V. d. S.; Taqueda, M. E. S.; de Paiva, J. L.; de Moraes, M. S.; de Moraes, D., Nusselt's correlations in agitated tanks using the spiral coil with Rushton turbine and PBT 45° impeller. Comparison with tanks containing vertical tube baffles. *Applied Thermal Engineering* **2017,** 110, 1331-1342.

21. Cheng, N.-S., Formula for the viscosity of a glycerol−water mixture. *Ind. Eng. Chem. Res.* **2008,** 47, (9), 3285-3288.

22. Righetti, M.; Salvetti, G.; Tombari, E., Heat capacity of glycerol from 298 to 383 K. *Thermochimica acta* **1998,** 316, (2), 193-195.

23. Bioucas, F. E. B.; Koller, T. M.; Fröba, A. P., Thermal conductivity of glycerol at atmospheric pressure between 268 K and 363 K by using a steady-state parallel-plate instrument. *International Journal of Thermophysics* **2024,** 45, (4), 52.

24. Ariba, H.; Wang, Y.; Devouge-Boyer, C.; Stateva, R. P.; Leveneur, S., Physicochemical properties for the reaction systems: Levulinic acid, its esters, and γ-valerolactone. *Journal of Chemical & Engineering Data* **2020,** 65, (6), 3008-3020.

25. The Engineering ToolBox, Heat capacity. In The Engineering ToolBox: 2024.

26. Altuntepe, E.; Emel'yanenko, V. N.; Forster-Rotgers, M.; Sadowski, G.; Verevkin, S. P.; Held, C., Thermodynamics of enzyme-catalyzed esterifications: II. Levulinic acid esterification with short-chain alcohols. *Appl Microbiol Biotechnol* **2017,** 101, (20), 7509-7521.

27. Pankajakshan, A.; Bawa, S. G.; Gavriilidis, A.; Galvanin, F., Autonomous kinetic model identification using optimal experimental design and retrospective data analysis: methane complete oxidation as a case study. *React. Chem. Eng.* **2023,** 8, (12), 3000-3017.

28. Asprey, S. P.; Macchietto, S., Statistical tools for optimal dynamic model building. *Computers & Chemical Engineering* **2000,** 24, (2-7), 1261-1267.

29. Reinhardt, H. E., Nonlinear parameter estimation (Yonathan Bard). *SIAM Rev.* **1975,** 17, (4), 703-704.
